# Trends in the Incidence and DALYs of Urolithiasis From 1990 to 2019: Results From the Global Burden of Disease Study 2019

**DOI:** 10.3389/fpubh.2022.825541

**Published:** 2022-03-04

**Authors:** Shasha Li, Xueying Huang, Jie Liu, Suru Yue, Xuefei Hou, Liren Hu, Jiayuan Wu

**Affiliations:** ^1^Clinical Research Service Center, Affiliated Hospital of Guangdong Medical University, Zhanjiang, China; ^2^Collaborative Innovation Engineering Technology Research Center of Clinical Medical Big Data Cloud Service in Medical Consortium of West Guangdong Province, Zhanjiang, China

**Keywords:** urolithiasis (urinary stones), incidence, disability-adjusted life years (DALYs), secular trend, Global Burden of Disease (GBD)

## Abstract

**Objectives:**

To provide a comprehensive assessment of the estimated burden and trend of urolithiasis at the global, regional, and national levels.

**Methods:**

The age-standardized rates (ASRs) of the incidence and disability-adjusted life years (DALYs) of urolithiasis from 1990 to 2019 were obtained from the Global Burden of Disease Study 2019 database. Estimated annual percentage changes (EAPCs) were calculated to quantify the temporal trends in urolithiasis burden.

**Results:**

In 2019, the ASRs of the incidence and DALYs were 1,394.03/100,000 and 7.35/100,000, respectively. The ASRs of the incidence and DALYs of urolithiasis decreased from 1990 to 2019 with EAPCs of −0.83 and −1.77, respectively. Males had a higher burden of urolithiasis than females. In 2019, the highest burden of urolithiasis was observed in regions with high–middle sociodemographic index (SDI), particularly in Eastern Europe, Central Asia, and Southeast Asia. The burden of urolithiasis increased in most countries or territories. The burden of urolithiasis and SDI had a non-linear relationship, and the estimated value of urolithiasis burden was the highest when the SDI value was ~0.7.

**Conclusion:**

Globally, the ASRs of the incidence and DALYs of urolithiasis decreased from 1990 to 2019, but an increasing trend was observed among many countries. More effective and appropriate medical and health policies are needed to prevent and early intervene in urolithiasis.

## Introduction

Urolithiasis, which is the formation of calculi or stones in the urinary tract, is a common urinary system disease that affects 10–15% of the world's population (1). The symptoms and high recurrence rate of urolithiasis greatly affect the quality of life of patients and increased the risk of comorbidities, such as fractures, renal dysfunction, obesity, diabetes, and cardiovascular diseases (2). Moreover, a study showed that the estimated cost of urolithiasis treatment in the United States was $3.79 billion in 2007 and is expected to increase by $1.24 billion/year by 2030 (3). Urolithiasis has become a great burden on public health.

Urolithiasis is a multifactorial disease influenced by diet, lifestyle, environment, and genetics. Scientific evidence agrees with the harmful role of high meat/animal protein intake and low-calcium diets in stone formation and the protective role of fruits and vegetable intake, balanced intake of low-fat dairy products, high fluid intake with a preference for strong tea, and physical exercise (4). Weight gain, obesity, and diabetes have been proven to be related to a higher risk of stone formation in several large prospective studies (5). Calcium oxalate stones are the most common type of urinary tract stones, which are found to be the result of certain enzyme deficiencies (6). Moreover, the occurrence of urolithiasis is related to climatic and geological factors (7). The epidemiology of urolithiasis may have changed dramatically in recent decades. However, up-to-date, comprehensive and accurate information on the burden of urolithiasis are still lacking. Sound and up-to-date evidence at the national level is essential to reflect the impact of public health policies and the provision of health care (8).

Studies that used point estimation rather than secular trend analysis to quantify the burden of urolithiasis are limited. In addition, the relationship between urolithiasis burden and economic development in different regions has not been studied. The Global Burden of Disease (GBD) study, which is now housed in the Institute for Health Metrics and Evaluation (IHME), has become a very important tool to global health governance since it was first published in the 1993 World Development Report (9). The GBD 2019 provides a good platform to integrate newly available data sets and enhance method performance and standardization to understand disease burden and its secular trend (8). The GBD 2019 contains the latest epidemiological data on 369 diseases and injuries in 204 countries or territories from 1990 to 2019, providing an opportunity to understand the burden of urolithiasis worldwide (8). In the GBD study, the burden of disease can be measured by incidence, prevalence, death, and disability-adjusted life years (DALYs). A systematic analysis comprehensively reporting the variation trends of urolithiasis helps decision makers reasonably formulate policies and allocate limited resources. In this study, we used the GBD 2019 data to reveal the secular trends in the incidence and disability-adjusted life years (DALYs) of urolithiasis at the global, regional, and national levels. Furthermore, we also determined the relationship between the burden of urolithiasis and the level of development as measured by the Human Development Index (HDI).

## Methods

We extracted the age-standardized rates (ASRs) of the incidence and DALYs of urolithiasis at the global, regional, and national levels from 1990 to 2019 from the Global Health Data Exchange website (http://ghdx.healthdata.org/gbd-results-tool). The GBD estimation process is based on identifying multiple relevant data sources for each disease or injury, including censuses, household surveys, civil registration and vital statistics, disease registries, health service use, air pollution monitors, satellite imaging, disease notification, and other sources. The general methods used in the GBD 2019 were described in detail on the official website (http://www.healthdata.org/gbd/). DALYs, which is a standard indicator used to quantify burden, was calculated by adding years of life lost (YLL) and years lived with disability (YLD) (8). YLL represents the fatal component of the burden and takes into account the life expectancy and number of deaths from specific causes. YLD represents the non-fatal component of the burden and includes disease prevalence and the impact of the disease on disability. Urolithiasis is coded as N20–N23.0 in the 10th revision of the International Classification of Diseases. We compared the burden estimates of urolithiasis by sex and age groups separately. The age stratification in the GBD 2019 were as follows: 5-year age group from age 0–95 and then a single category for >95 years old.

Sociodemographic index (SDI) is a comprehensive indicator of the development level of a region or country, which is scored from 0 to 1 by calculating the gross domestic product per capita, mean education for those 15 years old and older, and total fertility rate for those under 25 years old (8). SDI values of all countries from 1990 to 2019 were obtained from Institute for Health Metrics and Evaluation. Based on the SDI values, the 204 countries or territories were divided into five SDI regions: low, low–middle, middle, high–middle, and high. The world was also geographically divided into 21 regions to observe for geographic disparities.

The Human Development Index (HDI) is a compound index based on three dimensions of life expectancy, education, and decent standards of living (10). HDI combines economic and social indicators to reveal the imbalance between economic growth and social development, and points out the comprehensive development of human health and longevity, access to education, living standards, living environment, and degree of freedom. HDI is an important index to measure the comprehensive national strength of a country, and can be collected from the website of World Bank (http://hdr.undp.org/en/content/human-development-index-hdi/). To explore the influential factors for EAPCs, we evaluated the relationship between EAPC and ASR in 1990 and HDI in 2019 at the national level through scatter plots and Pearson correlation analysis. The ASR for urolithiasis in 1990 shows the baseline disease database, and the HDI in 2019 can be used as a surrogate indicator of the quality and availability of healthcare in each country.

ASRs (rates per 100,000 population) were calculated by summing up the products of the age-specific rates (*a*_*i*_, where *i* is the *i*th age class) and the number of persons (or the weight, *w*_*i*_) in the same age subgroup *i* of the selected reference standard population and then dividing the sum of the standard population weights (11). The ASRs and 95% uncertainty intervals (UIs) were calculated based on the GBD 2019 global age standard population.


(1) $$\begin{array}{r} ASR\;=\;\frac{\sum_{i=1}^{A}a_{i}w_{i}}{\sum_{i=1}^{A}w_{i}}\times 100000 \end{array}$$

Estimated annual percentage change (EAPC) is an indicator commonly used to reflect the ASR trends over a specified time period. The natural logarithm of the regression-line fit to ASR is *y* = *a* + *bx* + *e*, where *x* = the calendar year. EAPC is calculated as 100 × [exp(*b*) – 1], and its 95% UI can also be obtained from a linear regression model (12). If the estimated EAPC and the lower bound of its 95% UI are >0, then ASRs would exhibit an increasing trend; if the estimated EAPC and the upper bound of its 95% UI are <0, then ASRs would exhibit a downward trend; otherwise, the ASRs would be considered stable.

All analyses were conducted using R program (version 4.0.5, R Core Team). *P* < 0.05 was considered statistically significant.

## Results

### Burden of Urolithiasis at the Global Level

**Figure 1:**
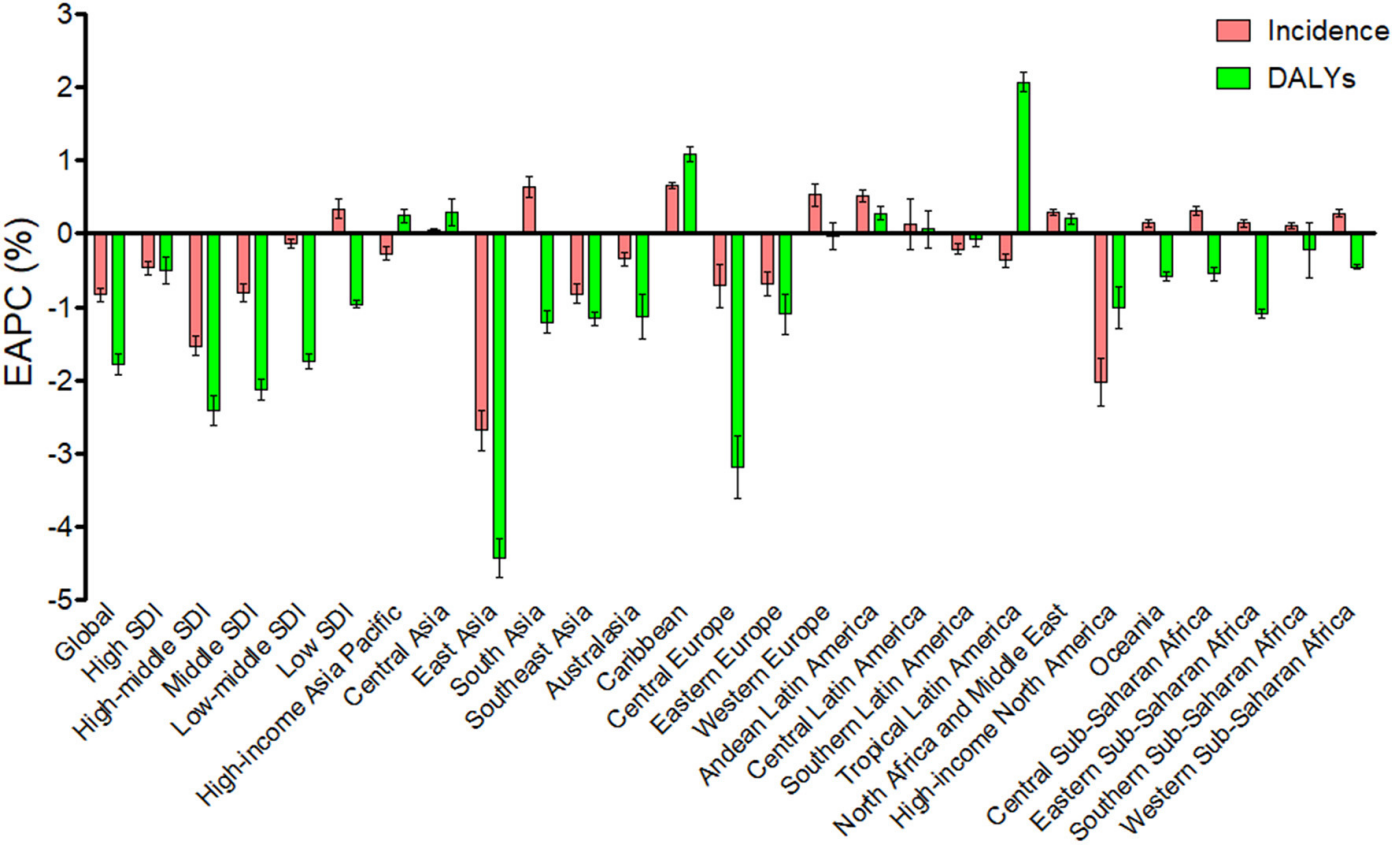
The EAPC of age-standardized incidence and DALYs rates of urolithisis at the global and regional levels.

The burden of urolithiasis and its trends at the global and regional levels are listed in Table 1. Globally, the ASR of urolithiasis incidence decreased from 1,696.18 (95% UI: 1,358.11–2,078.11) in 1990 to 1,394.03 (95% UI: 1,126.4–1,688.16) per 100,000 population in 2019 with an EAPC of −0.83 (95% UI: −0.92 to −0.74). The age-standardized DALY rate of urolithiasis also decreased from 11.75 (95% UI: 8.57–14.39) in 1990 to 7.35 (95% UI: 5.82–9.04) per 100 000 population in 2019 with an EAPC of −1.77 (95% UI: −1.92 to −1.63; Table 1; Figure 1).

**Table 1:** Age-standardized rates of incidence and disability-adjusted life years of urolithiasis in 2019 and their temporal trend from 1990 to 2019 at the global and regional levels.

	**Incidence (95% UI)**			**DALYs (95% UI)**		
	**ASR in 1990** **(per 100,000 population)**	**ASR in 2019** **(per 100,000 population)**	**EAPC (1990–2019)**	**ASR in 1990** **(per 100,000 population)**	**ASR in 2019** **(per 100,000 population)**	**EAPC (1990–2019)**
**Global**	1,696.18 (1,358.11–2,078.11)	1,394.03 (1,126.4–1,688.16)	−0.83 (−0.92 to −0.74)	11.75 (8.57–14.39)	7.35 (5.82–9.04)	−1.77 (−1.92 to −1.63)
**Sex**						
Male	2,353.15 (1,878.96–2,879.17)	1,856.87 (1,495.27–2,245.34)	−1.01 (−1.10 to −0.91)	14.76 (9.92–18.82)	9.10 (6.92–11.34)	−1.83 (−1.94 to −1.71)
Female	1,066.85 (851.17–1,305.09)	947.22 (761.21–1,148.43)	−0.47 (−0.59 to −0.36)	9.11 (6.73–10.91)	5.72 (4.58–7.07)	−1.76 (−1.97 to −1.55)
**Socio-demographic index**						
High SDI	1,556.68 (1,228.01–1,924.36)	1,288.65 (1,053.86–1,544.09)	−0.47 (−0.56 to −0.38)	6.68 (5.07–8.53)	5.30 (3.99–6.75)	−0.50 (−0.69 to −0.31)
High-middle SDI	2,273.31 (1,819.79–2,776.75)	1,576.44 (1,268.89–1,918.41)	−1.53 (−1.66 to −1.40)	14.68 (11.99–17.61)	7.94 (6.33–9.79)	−2.41 (−2.62 to −2.21)
Middle SDI	1,582.66 (1,255.22–1,938.68)	1,242.73 (1,000.94–1,510.56)	−0.81 (−0.94 to −0.68)	12.91 (8.23–16.13)	7.49 (5.81–9.17)	−2.12 (−2.26 to −1.98)
Low-middle SDI	1,485.55 (1,193.43–1,813.78)	1,460.63 (1,159.28–1,788.45)	−0.13 (−0.20 to −0.07)	13.86 (7.44–18.79)	8.85 (6.24–11.25)	−1.74 (−1.83 to −1.64)
Low SDI	954.92 (755.16–1,176.74)	981.88 (771.35–1,212.27)	0.34 (0.21 to 0.47)	7.60 (5.21–10.43)	5.88 (4.33–7.87)	−0.96 (−1.01 to −0.91)
**Geographic region**						
High-income Asia Pacific	1,536.37 (1,181.32–1,920.86)	1,475.15 (1,172.93–1,795.88)	−0.27 (−0.35 to −0.18)	5.48 (3.91–7.35)	5.70 (4.18–7.45)	0.25 (0.16 to 0.34)
Central Asia	1,755.54 (1,403.55–2,151.02)	1,787.98 (1,435.54–2,174.91)	0.05 (0.02 to 0.08)	10.83 (8.05–14.57)	12.42 (9.88–15.89)	0.29 (0.12 to 0.47)
East Asia	1,592.83 (1,245.33–1,984.66)	901.81 (727.27–1,088.77)	−2.68 (−2.96 to −2.40)	16.61 (9.81–20.36)	5.35 (4.17–6.69)	−4.43 (−4.69 to −4.17)
South Asia	1,518.02 (1,206.38–1,880.74)	1,757.72 (1,382.75–2,184.63)	0.64 (0.49 to 0.79)	11.47 (7.08–16.51)	8.33 (6.00–11.10)	−1.20 (−1.36 to −1.05)
Southeast Asia	1,904.34 (1,511.83–2,313.01)	1,652.63 (1,348.20–1,979.06)	−0.82 (−0.95 to −0.69)	17.17 (7.93–23.59)	13.06 (6.92–16.54)	−1.15 (−1.25 to −1.06)
Australasia	1,405.30 (1,096.40–1,739.12)	1,283.37 (1,004.68–1,573.74)	−0.35 (−0.44 to −0.26)	7.78 (6.25–9.65)	5.37 (4.04–6.95)	−1.13 (−1.42 to −0.83)
Caribbean	1,056.51 (830.22–1,310.52)	1,239.73 (979.28–1,540.36)	0.66 (0.63 to 0.70)	6.61 (5.13–8.30)	8.14 (6.26–10.45)	1.08 (0.98 to 1.18)
Central Europe	1,657.22 (1,324.62–2,032.82)	1,178.91 (977.08–1,400.97)	−0.71 (−1.00 to −0.42)	13.51 (11.06–18.26)	4.52 (3.40–5.95)	−3.18 (−3.60 to −2.76)
Eastern Europe	5,143.77 (4,155.80–6,201.33)	4,433.72 (3,542.49–5,414.66)	−0.69 (−0.85 to −0.53)	29.44 (24.01–35.53)	23.61 (18.69–29.23)	−1.09 (−1.37 to −0.82)
Western Europe	1,490.85 (1,183.00–1,846.74)	1,490.21 (1,181.37–1,829.24)	0.53 (0.38 to 0.69)	6.55 (5.04–8.41)	5.55 (3.99–7.28)	−0.04 (−0.21 to 0.14)
Andean Latin America	1,609.50 (1,290.27–1,977.80)	1,772.43 (1,472.60–2,110.69)	0.52 (0.44 to 0.60)	6.32 (4.46–8.49)	6.40 (4.62–8.44)	0.28 (0.19 to 0.37)
Central Latin America	974.87 (774.06–1,202.20)	1,012.43 (810.44–1,222.61)	0.13 (−0.22 to 0.49)	8.21 (6.86–9.78)	8.24 (6.70–10.57)	0.06 (−0.20 to 0.32)
Southern Latin America	1,646.58 (1,275.16–2,070.50)	1,674.47 (1,295.15–2,119.25)	−0.21 (−0.28 to −0.14)	5.23 (3.53–7.30)	5.54 (3.78–7.74)	−0.07 (−0.17 to 0.02)
Tropical Latin America	1,034.66 (833.81–1,253.92)	969.93 (789.43–1,165.79)	−0.36 (−0.45 to −0.28)	5.43 (4.34–6.76)	8.55 (6.84–12.13)	2.07 (1.93 to 2.20)
North Africa and Middle East	1,159.44 (904.44–1,445.58)	1,250.71 (985.87–1,553.28)	0.29 (0.25 to 0.33)	3.95 (2.73–5.46)	4.10 (2.82–5.65)	0.20 (0.14 to 0.27)
High–income North America	1,621.04 (1,270.22–2,010.69)	982.95 (843.80–1,137.38)	−2.02 (−2.34 to −1.69)	6.16 (4.55–8.08)	4.52 (3.59–5.63)	−1.01 (−1.29 to −0.72)
Oceania	978.39 (758.56–1,220.19)	1,033.10 (799.54–1,296.18)	0.14 (0.09 to 0.19)	7.31 (3.99–10.28)	6.25 (3.86–8.80)	−0.58 (−0.64 to −0.52)
Central Sub-Saharan Africa	533.21 (417.75–663.53)	575.37 (446.63–711.22)	0.31 (0.24 to 0.37)	5.02 (3.11–7.83)	4.33 (2.77–6.34)	−0.54 (−0.64 to −0.45)
Eastern Sub-Saharan Africa	548.62 (431.96–674.35)	565.68 (444.29–692.31)	0.15 (0.09 to 0.20)	6.37 (4.24–10.05)	4.83 (3.18–7.50)	−1.09 (−1.16 to −1.03)
Southern Sub-Saharan Africa	701.37 (552.78–863.37)	725.49 (574.19–893.26)	0.11 (0.07 to 0.15)	3.71 (2.85–4.68)	3.46 (2.63–4.44)	−0.22 (−0.60 – 0.15)
Western Sub-Saharan Africa	688.96 (543.93–847.52)	735.82 (579.31–902.54)	0.28 (0.22 to 0.34)	4.12 (2.55–5.79)	3.61 (2.42–4.79)	−0.45 (−0.49 to −0.42)

*DALYs, disability-adjusted life years; ASR, age-standardized rate; EAPC, estimated annual percentage change; UI, uncertainty interval*.

The ASRs of the incidence and DALY of urolithiasis were higher in males than in females during the observed period. In 2019, the ASRs of urolithiasis incidence were 1,856.87 (95% UI: 1,495.27–2,245.34) and 947.22 (95% UI: 761.21–1,148.43) per 100,000 population for males and females, respectively. The age-standardized DALY rates was 9.10 (95% UI: 6.92–11.34) and 5.72 (95% UI: 4.58–7.07) per 100 000 population for males and females, respectively. A greater decrement in the ASRs of the incidence and DALY of urolithiasis was found in males than females from 1990 to 2019 (Table 1).

In 2019, the ASRs for the incidence and DALY of urolithiasis were the highest in the high–middle and low–middle SDI regions, respectively. The age-standardized incidence rate of urolithiasis in low SDI regions showed an increasing trend; and the age-standardized DALY rate in all SDI regions showed a decreasing trend from 1990 to 2019. The ASRs of urolithiasis had the largest decrease in the high–middle SDI regions (Table 1; Figure 1).

**Figure 2:**
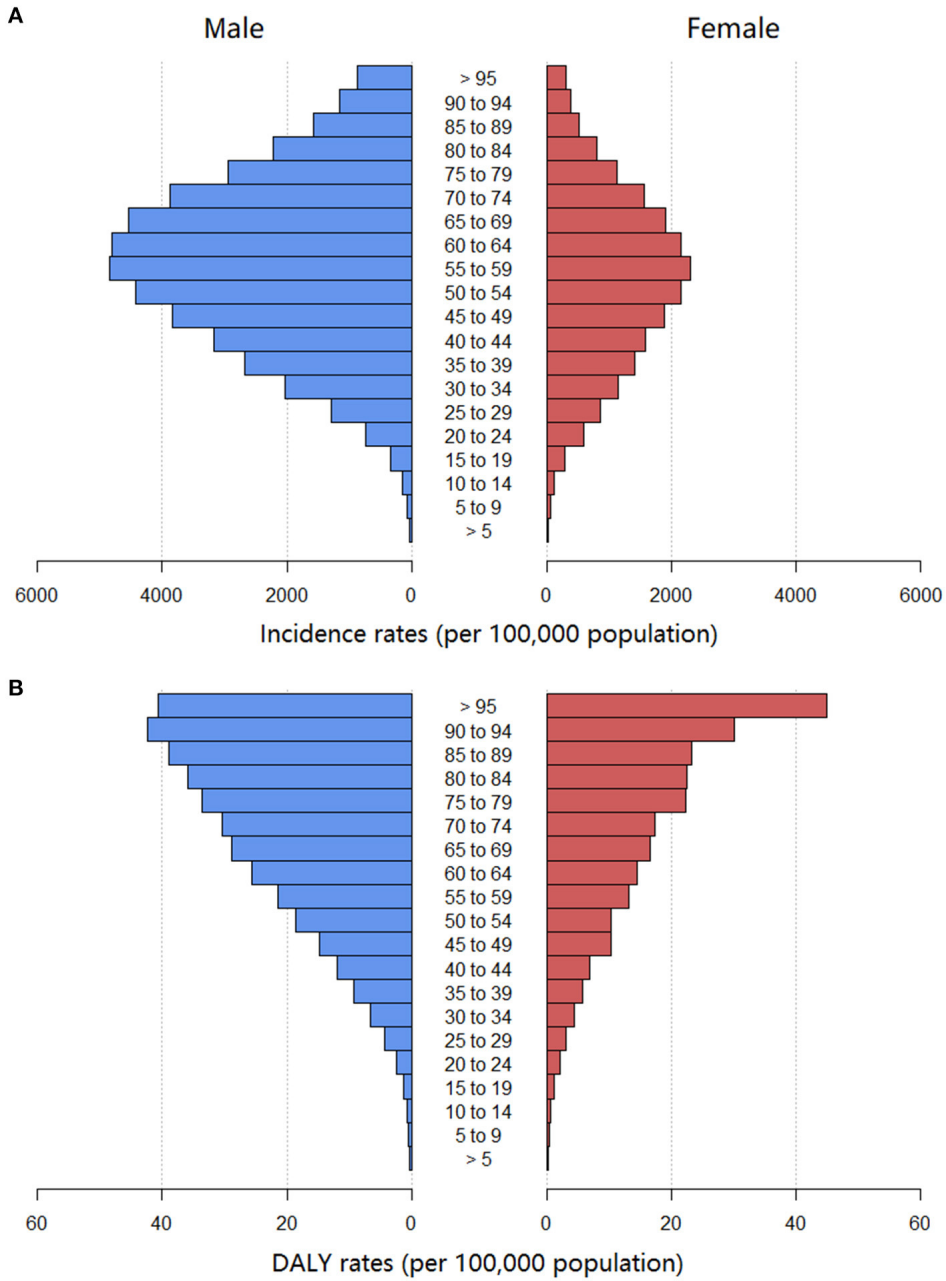
The age-standardized incidence rate **(A)** and DALYs rate **(B)** of urolithiasis of different age groups globally. DALYs, disability-adjusted life years.

We also analyzed the incidence rates and DALY rates of urolithiasis for males and females in different age groups (Figure 2). The incidence rates and DALY rates were higher for males than for females in all age groups with the exception of the DALY rates in the aged > 95 years. The highest incidence rates were in the age of 55–59 years. The DALY rates increased with age, and the highest DALY rates were in the population aged > 95 years.

### Burden of Urolithiasis at the Regional Level

In 2019, the highest age-standardized incidence rates were in Eastern Europe (4,433.72, 95% UI: 3,542.49–5,414.66 per 100,000 population) and Central Asia (1,787.98, 95% UI: 1,435.54–2,174.91 per 100,000 population). Similarly, the highest age-standardized DALY rates were in Eastern Europe (23.61, 95% UI: 18.69–29.23 per 100,000 population), followed by Southeast Asia and Central Asia. The change trend of the annual ASR of urolithiasis differed considerably among the 21 geographic regions. As shown in Figure 1, the ASRs for the incidence and DALYs of urolithiasis increased in the Caribbean, Andean Latin America, Central Asia, North Africa, and Middle East in the observed period, and the most remarkable increase was noted in the Caribbean for incidence (EAPC = 0.66, 95% UI: 0.63–0.7) and in the Tropical Latin America for DALYs (EAPC = 2.07, 95% UI: 1.93–2.2). The ASRs for incidence and DALYs decreased in East Asia, Southeast Asia, high-income North America, Australasia, Central Europe, and Eastern Europe, and the most remarkable decrease was in East Asia (EAPC = −2.68, 95% UI: −2.96 to −2.4 for incidence; EAPC = −4.43, 95% UI: −4.69 to −4.17 for DALYs).

### Burden of Urolithiasis at the National Level

**Figure 3:**
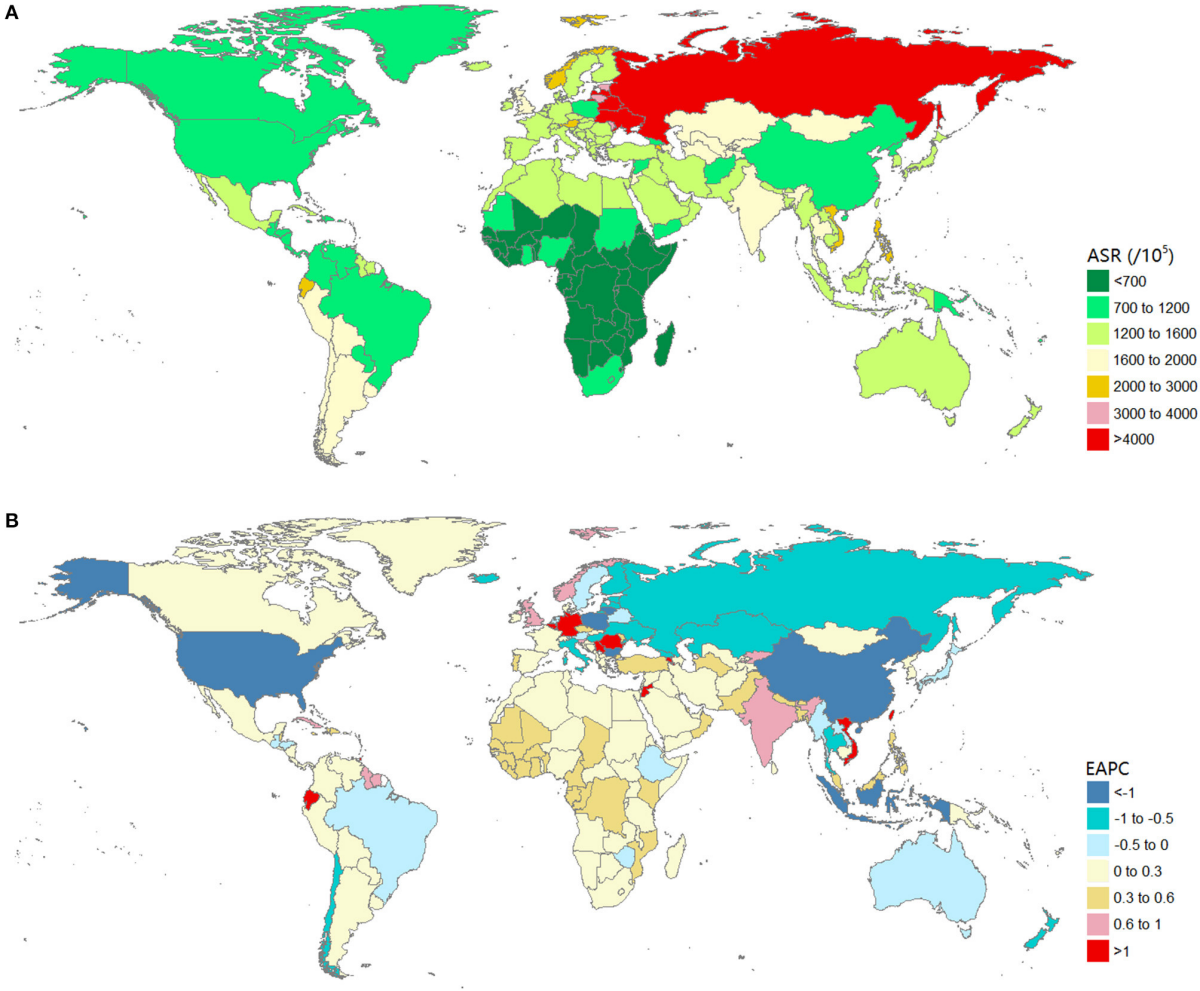
The global burden of urolithiasis in 204 countries or territories. **(A)** The age-standardized incidence rate of urolithiasis in 2019. **(B)** The EAPC of age-standardized prevalence rate of urolithiasis from 1990 to 2019. Countries with an extreme number of cases/evolution were annotated. EAPC, estimated annual percentage change.

**Figure 4:**
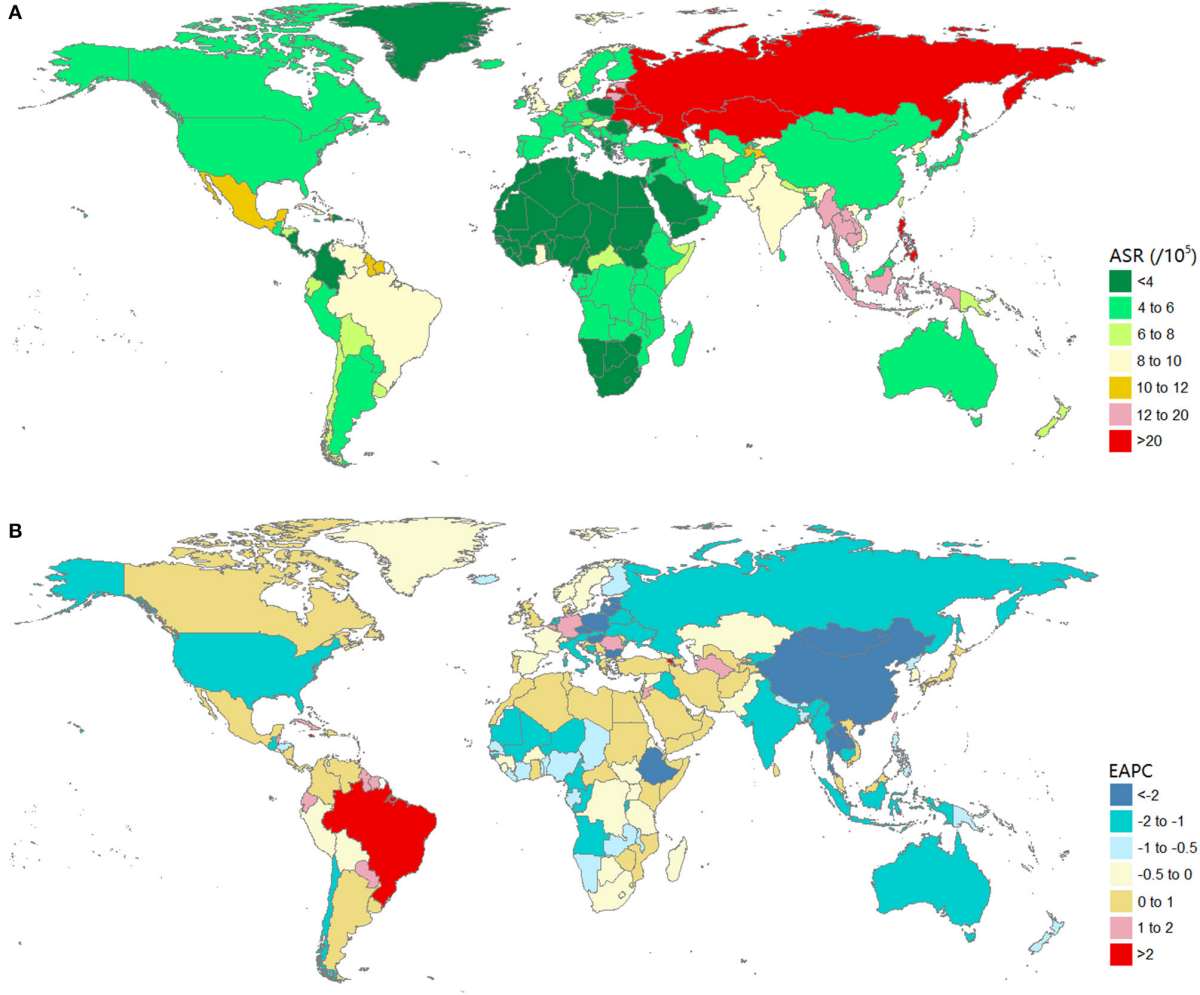
The global burden of urolithiasis in 204 countries or territories. **(A)** The age-standardized DALYs rate of urolithiasis in 2019. **(B)** The EAPC of age-standardized DALYs rate of urolithiasis from 1990 to 2019. Countries with an extreme number of cases/evolution were annotated. DALYs, disability-adjusted life years; EAPC, estimated annual percentage change.

The burden of urolithiasis and its trends in 204 countries or territories are shown in Supplementary Table 1. In 2019, the Russian Federation had the highest age-standardized incidence rates (4,541.88, 95% UI: 3,648.94–5,522 per 100,000 population), followed by Ukraine, Latvia, and Belarus (Figure 3A). Armenia had the highest age-standardized DALY rates (33.33, 95% UI: 21.71–61.27 per 100,000 population), followed by the Russian Federation, Philippines, Ukraine, Kazakhstan, Latvia, and Belarus (Figure 4A). The ASRs of the incidence and DALYs of urolithiasis increased among 156 and 75 countries or territories, respectively. The greatest increase in age-standardized incidence rates was found in Jordan, Romania, and Germany, whereas the most remarkable decrease was observed in Poland, China, Indonesia, and the United States of America (Figure 3B). The largest increase in age-standardized DALY rates was in Trinidad and Tobago, Armenia, and Jamaica, and the most remarkable decrease was observed in Poland, Bulgaria, and China (Figure 4B).

### Relationship Between Estimated Burden of Urolithiasis and SDI Level

**Figure 5:**
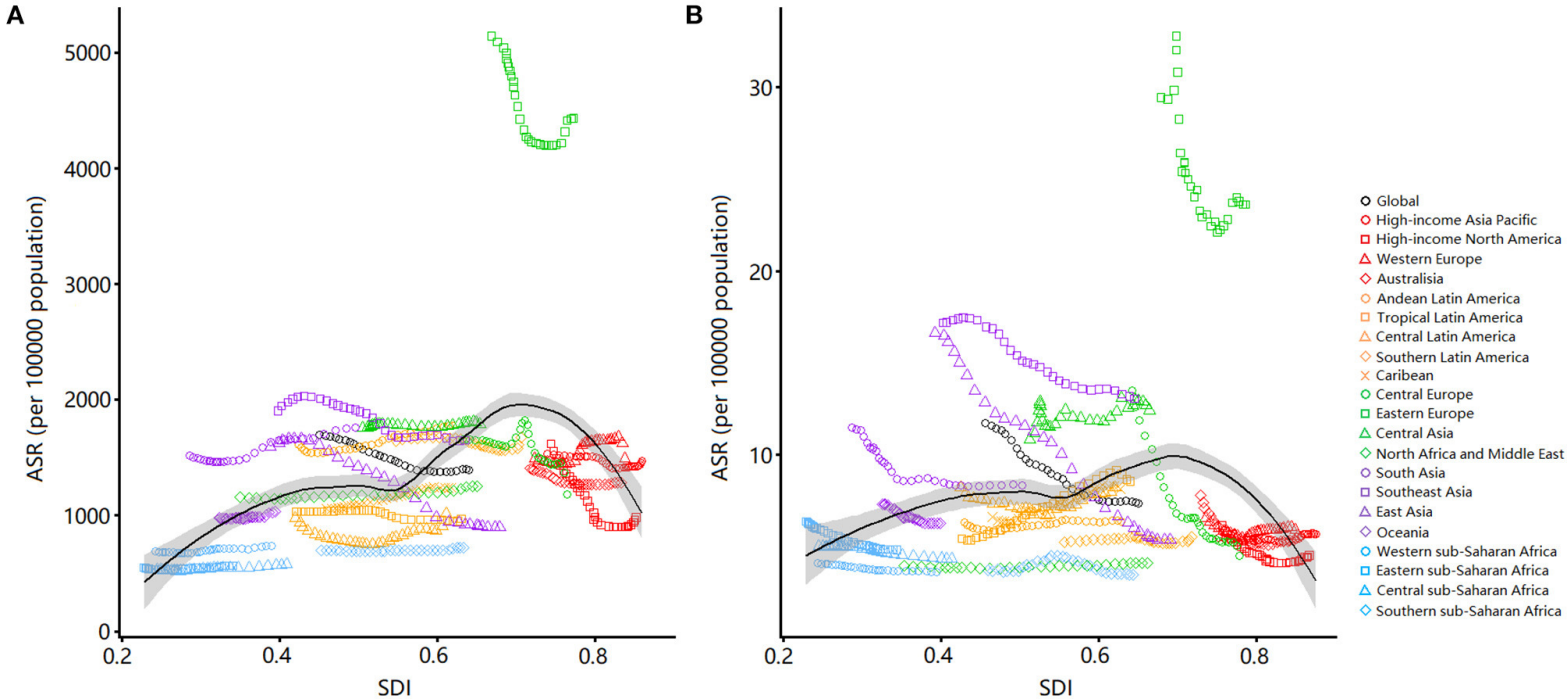
Age-standardized rates of incidence **(A)** and DALYs **(B)** for urolithiasis by SDI, 1990–2019, and expected value-based SDI. Age-standardized rates are plotted for 21 geographic regions between 1990 and 2019 against their SDIs. Points in each line from left to right represents the values from 1990 to 2019. The black line represents the average expected relationship between SDI and incidence **(A)** or DALYs **(B)** for urolithiasis based on values from all countries over the 1990–2019 estimation period. DALYs, disability-adjusted life years; SDI, sociodemographic index.

We clarified the association between the estimated burden of urolithiasis and the level of SDI in 21 geographic regions from 1990 to 2019 (Figure 5). The estimated burden of urolithiasis increased with SDI, reached a maximum at about 0.7, and then decreased with the further increase in SDI.

### Influence Factors for EAPC

**Figure 6:**
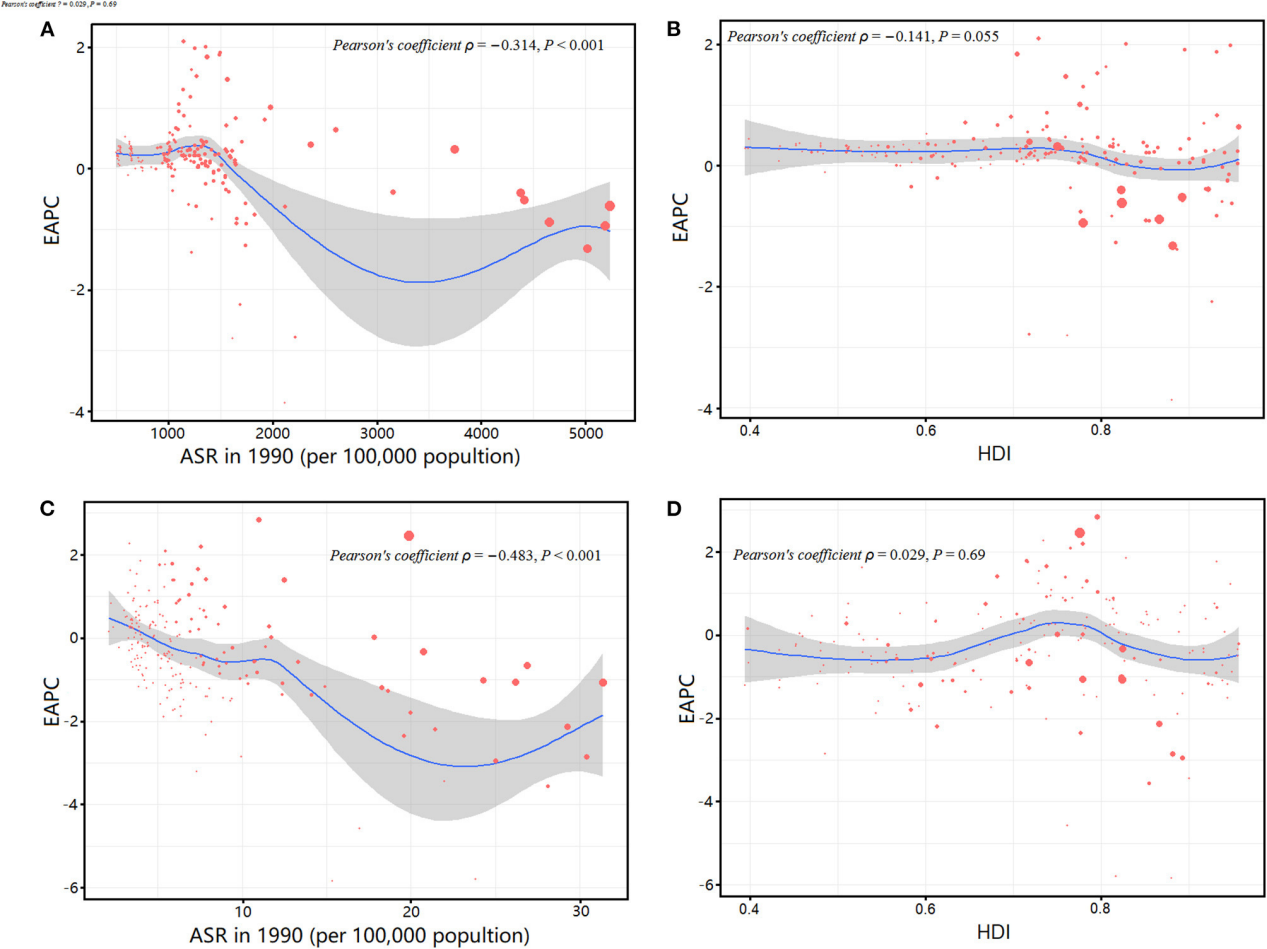
Correlation between the EAPC of age-standardized incidence rates and the age-standardized prevalence rates in 1990 **(A)**; the EAPC of age-standardized incidence rates and the HDIs in 2019 **(B)**; the EAPC of age-standardized DALY rates and the age-standardized DALY rates in 1990 **(C)**; and the EAPC of age-standardized DALY rates and the HDIs in 2019 **(D)**. The size of circle represents the age-standardized rates of incidence or DALYs in this country or territory in 2019. DALYs, disability-adjusted life years; EAPC, estimated annual percentage change; HDI, human development index.

We analyzed the relationships between EAPCs and ASRs in 1990 and HDI in 2019 in 204 countries or territories (Figure 6). The ASRs in 1990 were negatively correlated with the EAPCs of age-standardized incidence (ρ = −0.314, *P* < 0.001, Figure 6A) and DALY rates (ρ = −0.483, *P* < 0.001, Figure 6C), respectively. The HDI in 2019 and the EAPCs had no correlation with respect to the incidence (ρ = −0.141, *P* = 0.055, Figure 6B) and DALY rates (ρ = 0.029, *P* = 0.691, Figure 6D) of urolithiasis.

## Discussion

This study based on GBD 2019 aimed to conduct the first comprehensive assessment of the estimated burden and trend of urolithiasis at the global, regional, and national levels from 1990 to 2019. Males seem to be more susceptible to urolithiasis than females, which is consistent with the results of several studies (2). The exact pathophysiology of sex differences in urolithiasis is still uncertain, but could be explained as follows. For dietary habits (13), males tend to drink more alcohol and coffee and eat more meat than females. In terms of physiology, testosterone can promote stone formation, whereas estrogen seems to inhibit stone formation by regulating the synthesis of 1,25-dihydroxy-vitamin D (14). However, different findings seem paradoxical. For example, some studies emphasize the protective effects of estrogen, whereas in others, estrogen replacement therapy in postmenopausal women appears to be a risk factor for urolithiasis (15). In the present study, the global incidence of urolithiasis in males and females peaks in the 55–59 age group and then declines. A cohort study from Korea showed that the highest incidence of urolithiasis was in the aged 50–54 years for males 55–59 years for females (16). In France, the highest number of urinary stones for males and females were observed in the 40–49 and 30–39 age groups, respectively (17). The age distribution of urolithiasis varies among countries but is mainly concentrated in the middle-aged and elderly people. Middle-aged people are prone to urolithiasis because of their occupational stress (heavy work, low fluid intake, high dehydration rate) and unhealthy lifestyle (staying up late or irregular diet) (18). Moreover, the crude DALY rate of urolithiasis increases with age. The risks of urolithiasis recurrence and comorbidities, as well as disease burden, increase with age; thus, the higher DALY in elderly patients is caused by premature death and disability.

Globally, the burden estimates of urolithiasis, as measured by incidence and DALYs, decreased globally during the observation period. This decreasing trend has been influenced by the trends in urolithiasis in some regions, particularly in populous East Asia. For example, as the most populous country in the world, China has experienced a remarkable decline. In the past 20 years, China's diet structure has greatly changed (the consumption of fruits and vegetables by children and adults is on the rise) (19). However, the decreasing trend in the global burden of urolithiasis is different from many previous studies, which reveal an increasing trend (20). Notably, the indicators of the incidence of urolithiasis in these studies tend to be the absolute number or the unadjusted crude rate, whereas our results refer to the ASRs, which are more accurate in estimating urolithiasis burden. Although the global trend of urolithiasis is declining, urolithiasis may pose a substantial burden and challenge due to the aging and the increase in life expectancy of the world's population.

The burden of urolithiasis in 2019, as measured by incidence and DALYs, is the highest in Eastern Europe, which is inseparable from the contribution of the Russian Federation. The high incidence of urolithiasis in the Russian Federation is closely related to carnivorous diet and the increase in the incidence of obesity and diabetes (21). The possible underlying mechanism of stone formation in people with diabetes and obesity might be that excessive uric acid is produced in the urine because of insulin resistance and increased fatty acid production; thus, uric acid stones are eventually formed (22). The Dietary Approaches to Stop Hypertension, which involves the intake of diet rich in fruits, vegetables, legumes, and nuts; moderate in low-fat dairy products; and low in animal protein, refined grains, and sweets, can remarkably reduce the risk of urinary stones (23). Therefore, the primary prevention of urolithiasis through dietary intervention is a public health measure with low cost and huge social impact. The burden of urolithiasis has shown an increasing trend in many countries, such as Jordan, Romania, Germany, Trinidad and Tobago, and Armenia. The increase in the incidence of urolithiasis may be related to the improvement of diagnostic methods, such as the widespread use of computed tomography (CT) and X-rays in clinical practice (21). Moreover, changes in diet (Westernized dietary habits and less fluid intake) and lifestyle have also played a role in the increased incidence of urolithiasis (18). Detailed diet management and prevention strategies to reduce obesity and diabetes may be effective measures.

The burden of urolithiasis varies across regions and countries with different SDI levels because of the different epidemiological characteristics of risk factors associated with urolithiasis. When the SDI is lower than 0.7, the estimated burden of urolithiasis increases with the increase in SDI. Socioeconomic level is an important factor that affects the epidemiology of urolithiasis. For example, in China, the temperature in the southern province, Taiwan, is higher, but the prevalence of urolithiasis among residents in northern areas is higher because of their higher socioeconomic status (24). Chronic metabolic disease dominated by high calorie intake is more common in people with higher living standards (25). In addition, people from economically underdeveloped regions have fewer opportunities to obtain medical examinations, such as CT scan or ultrasound, which affects the detection rate of urolithiasis (18). Conversely, when the SDI is higher than 0.7, the estimated burden of urolithiasis decreases with the increase in SDI. Urolithiasis is now the second most expensive urological disease (20). Cost has gradually become an important factor in determining the best treatment for diseases (26). Underinsured patients will have a longer wait before undergoing surgery, and these delays may increase the risk of urinary tract infection, acute kidney injury, and potentially chronic kidney disease because the patient experiences persistent pain (27). Notably, the regions with an SDI value more than 0.7 usually have a relatively complete medical and health system and a more balanced and extensive distribution of medical resources, which greatly reduce the loss of healthy life for patients with urolithiasis. Moreover, the improvement of people's health awareness is helpful to the early detection and early treatment of urinary stones.

It is well known that urolithiasis is closely related to diet. A correct and balanced diet can effectively prevent the occurrence of stones. High fluid intake is an important factor in preventing kidney stone disease, with a 13% reduction in stone risk per 200 ml of water (28). Use of dietary plants and plant phenols also plays an important role in the prevention and treatment of stones (29). Patients with urolithiasis are advised to reduce dietary protein and sodium intake. Moderate dietary salt restriction helps limit urinary calcium excretion and may contribute to primary and secondary prevention of urolithiasis (30). Compared with a low-calcium diet, a balanced consumption of dairy products can reduce the intestinal absorption and urinary excretion of oxalate, and has a protective effect on stone disease. In summary, an effective stone-protecting diet should be rich in fruits and vegetables, low in animal protein and salt, balanced in dairy intake, and adequate in fluid intake. It is important to use diet to prevent urolithiasis and reduce the burden.

The limitations of the GBD 2019 database itself inevitably affected this study. First, the estimates for GBD burden are the combination of different data sources, and the accuracy of the estimates largely depend on the quality and quantity of the data used in the modeling process. The accuracy of burden estimation in areas with scarce or missing data is doubtful. Second, data on the representative regions and ethnic groups, as well as age groups, of many countries are scarce. Sparse data limits the certainty of the estimation of time trends and age patterns. Finally, data at the regional level may be misleading because it obscures the diversity of the existing situation within countries.

In conclusion, our research shows that the global burden of urolithiasis showed a decreased trend between 1990 and 2019. However, urolithiasis will continue to cause huge losses to healthy lives, especially for the elderly and in regions with high–middle SDIs. Our findings emphasize that urolithiasis is still a global health problem and indicate that the variations in urolithiasis burden in terms of region, country, gender, and age should be considered when formulating global health goals. More effective and appropriate medical and health policies are needed to prevent and early treat urolithiasis.

## Data Availability Statement

The original contributions presented in the study are included in the article/Supplementary Material, further inquiries can be directed to the corresponding author/s.

## Author Contributions

SL and XHu collected the data. SL and JL analyzed the data. SY and XHo wrote the first draft of the paper. LH and JW contributed to conception and design and oversaw the research. All authors contributed to the study design, analysis, and interpretation of data.

## Conflict of Interest

The authors declare that the research was conducted in the absence of any commercial or financial relationships that could be construed as a potential conflict of interest.

## Publisher's Note

All claims expressed in this article are solely those of the authors and do not necessarily represent those of their affiliated organizations, or those of the publisher, the editors and the reviewers. Any product that may be evaluated in this article, or claim that may be made by its manufacturer, is not guaranteed or endorsed by the publisher.

## Abbreviations

ASR: age-standardized rate
DALYs: disability-adjusted life years
SDI: sociodemographic index
HDIs: human development indices
GBD: global burden of disease
EAPC: estimated annual percentage change
UI: uncertainty interval.
