# Supplementary material for: Trends in the Incidence and DALYs of Urolithiasis From 1990 to 2019: Results From the Global Burden of Disease Study 2019
Source: Front Public Health. 2022 Mar 4;10:825541. doi: 10.3389/fpubh.2022.825541 (PMC8931285; doi:10.3389/fpubh.2022.825541)
Supplement: Supplementary file 1 [file Table_1.docx]

**Supplementary Table 1. Age-standardized rates of incidence and disability-adjusted life years of urolithiasis in 2019 and their temporal trend from 1990 to 2019 at the national level**

|  | **Incidence (95% UI)** | | | **DALYs (95% UI)** | | |
| --- | --- | --- | --- | --- | --- | --- |
|  | **ASR in 1990**  **(per 100,000 population)** | **ASR in 2019**  **(per 100,000 population)** | **EAPC (1990‒2019)** | **ASR in 1990**  **(per 100,000 population)** | **ASR in 2019**  **(per 100,000 population)** | **EAPC (1990‒2019)** |
| Afghanistan | 1097.81 (856.58 ‒ 1366.24) | 1184.27 (927.24 ‒ 1467.36) | 0.28 (0.23 ‒ 0.34) | 3.97 (2.57 ‒ 6.06) | 4.67 (3.06 ‒ 6.94) | 0.76 (0.67 ‒ 0.85) |
| Albania | 1278.50 (1014.03 ‒ 1562.27) | 1284.26 (1027.10 ‒ 1572.40) | 0.02 (-0.02 ‒ 0.06) | 6.54 (3.86 ‒ 9.14) | 3.82 (2.47 ‒ 5.43) | -1.48 (-1.95 ‒ -1.02) |
| Algeria | 1142.03 (884.94 ‒ 1429.78) | 1214.32 (938.28 ‒ 1510.07) | 0.22 (0.18 ‒ 0.26) | 3.49 (2.22 ‒ 5.12) | 3.69 (2.39 ‒ 5.44) | 0.26 (0.21 ‒ 0.30) |
| American Samoa | 1035.56 (816.08 ‒ 1288.58) | 1038.32 (802.58 ‒ 1300.76) | -0.13 (-0.20 ‒ -0.06) | 7.83 (4.41 ‒ 11.16) | 4.93 (3.51 ‒ 6.60) | -2.32 (-3.05 ‒ -1.58) |
| Andorra | 1430.06 (1112.26 ‒ 1784.26) | 1417.66 (1105.13 ‒ 1764.58) | -0.04 (-0.07 ‒ -0.02) | 7.74 (4.67 ‒ 10.70) | 6.36 (4.18 ‒ 8.73) | -0.59 (-0.75 ‒ -0.43) |
| Angola | 538.34 (422.35 ‒ 665.29) | 574.59 (446.56 ‒ 710.03) | 0.27 (0.20 ‒ 0.34) | 6.33 (3.59 ‒ 10.92) | 4.24 (2.79 ‒ 6.60) | -1.65 (-1.79 ‒ -1.51) |
| Antigua and Barbuda | 1007.28 (775.5 ‒ 1244.96) | 1120.50 (878.39 ‒ 1394.75) | 0.43 (0.40 ‒ 0.47) | 4.47 (3.27 ‒ 5.94) | 6.04 (4.61 ‒ 7.76) | 1.14 (1.04 ‒ 1.24) |
| Argentina | 1646.15 (1267.08 ‒ 2085.16) | 1664.07 (1287.41 ‒ 2095.93) | 0.06 (0.03 ‒ 0.09) | 4.85 (3.19 ‒ 7.02) | 5.22 (3.42 ‒ 7.58) | 0.31 (0.26 ‒ 0.36) |
| Armenia | 1977.83 (1596.22 ‒ 2383.12) | 2540.63 (1939.79 ‒ 3808.42) | 1.01 (0.87 ‒ 1.15) | 19.88 (15.29 ‒ 25.86) | 33.33 (21.71 ‒ 61.27) | 2.46 (2.07 ‒ 2.85) |
| Australia | 1355.96 (1054.72 ‒ 1677.63) | 1282.28 (986.27 ‒ 1592.12) | -0.25 (-0.31 ‒ -0.18) | 7.29 (5.77 ‒ 9.08) | 5.01 (3.64 ‒ 6.62) | -1.18 (-1.45 ‒ -0.90) |
| Austria | 3156.38 (2860.30 ‒ 3443.99) | 2409.47 (1729.79 ‒ 3299.85) | -0.39 (-0.58 ‒ -0.21) | 14.9 (11.86 ‒ 18.70) | 7.92 (5.03 ‒ 12.18) | -1.18 (-1.53 ‒ -0.82) |
| Azerbaijan | 1561.18 (1224.2 ‒ 1926.40) | 1670.51 (1320.01 ‒ 2064.77) | 0.28 (0.21 ‒ 0.35) | 4.99 (3.28 ‒ 7.29) | 6.15 (4.00 ‒ 8.69) | 0.89 (0.70 ‒ 1.09) |
| Bahamas | 1014.65 (793.52 ‒ 1255.32) | 1110.49 (867.16 ‒ 1373.13) | 0.36 (0.34 ‒ 0.38) | 5.01 (3.83 ‒ 6.45) | 6.22 (4.70 ‒ 8.11) | 0.84 (0.71 ‒ 0.98) |
| Bahrain | 1202.04 (917.83 ‒ 1505.48) | 1292.07 (1004.2 ‒ 1626.35) | 0.32 (0.28 ‒ 0.36) | 3.72 (2.39 ‒ 5.37) | 4.20 (2.65 ‒ 6.30) | 0.50 (0.30 ‒ 0.70) |
| Bangladesh | 1330.70 (1044.12 ‒ 1667.32) | 1484.37 (1161.51 ‒ 1837.85) | 0.40 (0.35 ‒ 0.46) | 6.92 (4.64 ‒ 9.68) | 5.49 (3.73 ‒ 7.72) | -0.70 (-0.80 ‒ -0.60) |
| Barbados | 1067.59 (843.17 ‒ 1315.32) | 1196.00 (946.51 ‒ 1469.55) | 0.44 (0.38 ‒ 0.50) | 6.25 (4.85 ‒ 8.35) | 7.79 (5.75 ‒ 9.91) | 0.89 (0.74 ‒ 1.04) |
| Belarus | 4382.06 (3588.86 ‒ 5277.56) | 4017.99 (3181.04 ‒ 4977.84) | -0.40 (-0.51 ‒ -0.30) | 24.26 (19.17 ‒ 30.50) | 20.03 (14.50 ‒ 26.28) | -1.02 (-1.14 ‒ -0.89) |
| Belgium | 1486.33 (1151.45 ‒ 1866.12) | 1545.25 (1189.04 ‒ 1909.61) | 1.88 (1.02 ‒ 2.74) | 5.40 (3.82 ‒ 7.46) | 6.03 (4.14 ‒ 8.27) | 1.77 (1.04 ‒ 2.50) |
| Belize | 1038.72 (815.90 ‒ 1283.29) | 1195.09 (938.73 ‒ 1476.80) | 0.55 (0.50 ‒ 0.61) | 5.14 (3.82 ‒ 6.90) | 8.56 (6.71 ‒ 10.60) | 1.75 (1.31 ‒ 2.20) |
| Benin | 624.96 (491.76 ‒ 766.27) | 677.31 (530.21 ‒ 834.42) | 0.34 (0.26 ‒ 0.42) | 3.77 (2.52 ‒ 5.58) | 3.15 (2.25 ‒ 4.25) | -0.67 (-0.72 ‒ -0.62) |
| Bermuda | 1021.67 (791.76 ‒ 1272.93) | 1122.78 (873.55 ‒ 1400.88) | 0.33 (0.32 ‒ 0.35) | 4.57 (3.41 ‒ 5.97) | 5.00 (3.72 ‒ 6.59) | 0.33 (0.19 ‒ 0.48) |
| Bhutan | 1324.69 (1048.92 ‒ 1648.43) | 1504.04 (1184.36 ‒ 1864.77) | 0.45 (0.41 ‒ 0.50) | 8.71 (5.27 ‒ 15.25) | 6.91 (4.63 ‒ 10.56) | -0.86 (-0.95 ‒ -0.76) |
| Bolivia (Plurinational State of) | 1594.50 (1238.57 ‒ 2015.07) | 1661.89 (1284.24 ‒ 2086.54) | 0.18 (0.12 ‒ 0.24) | 8.93 (5.55 ‒ 12.76) | 7.80 (5.25 ‒ 10.64) | -0.36 (-0.46 ‒ -0.27) |
| Bosnia and Herzegovina | 1242.34 (988.97 ‒ 1507.91) | 1270.68 (1012.50 ‒ 1552.54) | 0.11 (0.08 ‒ 0.15) | 5.57 (3.94 ‒ 7.45) | 4.03 (2.67 ‒ 5.60) | -1.44 (-1.75 ‒ -1.12) |
| Botswana | 642.21 (505.79 ‒ 791.62) | 671.78 (520.67 ‒ 827.97) | 0.13 (0.04 ‒ 0.22) | 3.26 (2.21 ‒ 4.47) | 3.45 (2.29 ‒ 4.79) | 0.00 (-0.20 ‒ 0.20) |
| Brazil | 1036.90 (835.66 ‒ 1256.84) | 969.89 (790.08 ‒ 1165.75) | -0.38 (-0.46 ‒ -0.29) | 5.48 (4.40 ‒ 6.81) | 8.65 (6.91 ‒ 12.33) | 2.08 (1.94 ‒ 2.22) |
| Brunei Darussalam | 1538.37 (1199.75 ‒ 1905.92) | 1496.08 (1168.31 ‒ 1849.76) | -0.12 (-0.19 ‒ -0.06) | 10.80 (7.57 ‒ 15.13) | 8.48 (6.47 ‒ 11.00) | -0.56 (-0.79 ‒ -0.32) |
| Bulgaria | 1734.17 (1391.66 ‒ 2208.49) | 1267.09 (1013.20 ‒ 1537.38) | -1.27 (-1.54 ‒ -0.99) | 23.82 (18.25 ‒ 38.74) | 4.64 (3.27 ‒ 6.40) | -5.78 (-6.86 ‒ -4.70) |
| Burkina Faso | 633.41 (502.19 ‒ 773.91) | 686.69 (539.8 ‒ 844.24) | 0.34 (0.29 ‒ 0.39) | 4.10 (2.69 ‒ 6.06) | 3.95 (2.61 ‒ 5.76) | -0.22 (-0.30 ‒ -0.14) |
| Burundi | 514.89 (403.89 ‒ 631.71) | 525.01 (408.44 ‒ 646.90) | 0.08 (0.03 ‒ 0.12) | 5.66 (2.59 ‒ 10.91) | 4.34 (2.24 ‒ 7.26) | -1.25 (-1.39 ‒ -1.11) |
| Cabo Verde | 621.14 (493.82 ‒ 766.21) | 686.98 (540.00 ‒ 847.34) | 0.42 (0.34 ‒ 0.49) | 2.12 (1.46 ‒ 2.98) | 2.28 (1.50 ‒ 3.22) | 0.14 (0.07 ‒ 0.22) |
| Cambodia | 1397.70 (1137.76 ‒ 1667.82) | 1496.36 (1193.87 ‒ 1823.68) | 0.12 (0.09 ‒ 0.16) | 18.24 (4.95 ‒ 29.47) | 13.79 (4.77 ‒ 20.66) | -1.19 (-1.30 ‒ -1.08) |
| Cameroon | 636.33 (497.63 ‒ 781.18) | 685.95 (536.64 ‒ 844.09) | 0.31 (0.23 ‒ 0.39) | 4.24 (2.51 ‒ 6.51) | 3.34 (2.26 ‒ 4.70) | -1.01 (-1.10 ‒ -0.91) |
| Canada | 1006.97 (803.18 ‒ 1232.13) | 1057.58 (842.25 ‒ 1298.52) | 0.25 (0.22 ‒ 0.28) | 4.33 (3.26 ‒ 5.70) | 4.81 (3.68 ‒ 6.16) | 0.76 (0.58 ‒ 0.94) |
| Central African Republic | 532.32 (418.22 ‒ 660.14) | 569.1 (439.49 ‒ 699.28) | 0.29 (0.23 ‒ 0.35) | 6.92 (3.80 ‒ 12.01) | 7.11 (3.88 ‒ 12.64) | 0.15 (0.00 ‒ 0.30) |
| Chad | 625.77 (490.57 ‒ 768.12) | 697.84 (545.71 ‒ 858.78) | 0.44 (0.36 ‒ 0.53) | 3.95 (2.54 ‒ 5.96) | 3.36 (2.36 ‒ 4.64) | -0.66 (-0.73 ‒ -0.60) |
| Chile | 1650.14 (1270.17 ‒ 2085.88) | 1697.13 (1313.63 ‒ 2162.36) | -0.91 (-1.15 ‒ -0.67) | 6.36 (4.56 ‒ 8.53) | 6.11 (4.31 ‒ 8.37) | -1.09 (-1.35 ‒ -0.82) |
| China | 1614.23 (1262.27 ‒ 2011.52) | 889.90 (717.04 ‒ 1075.44) | -2.80 (-3.09 ‒ -2.51) | 16.92 (9.96 ‒ 20.73) | 5.27 (4.09 ‒ 6.66) | -4.57 (-4.85 ‒ -4.30) |
| Colombia | 744.48 (585.26 ‒ 915.81) | 767.44 (597.06 ‒ 949.94) | 0.14 (0.10 ‒ 0.18) | 3.53 (2.68 ‒ 4.56) | 3.97 (2.97 ‒ 5.28) | 0.90 (0.53 ‒ 1.28) |
| Comoros | 515.08 (405.39 ‒ 628.73) | 541.33 (422.81 ‒ 665.08) | 0.20 (0.17 ‒ 0.23) | 5.02 (2.40 ‒ 9.11) | 4.78 (2.52 ‒ 8.55) | -0.4 (-0.56 ‒ -0.25) |
| Congo | 534.73 (416.09 ‒ 662.13) | 581.49 (456.32 ‒ 722.66) | 0.35 (0.28 ‒ 0.41) | 5.83 (3.62 ‒ 10.16) | 4.47 (2.92 ‒ 6.73) | -1.22 (-1.39 ‒ -1.04) |
| Cook Islands | 995.66 (767.16 ‒ 1249.80) | 1030.71 (791.77 ‒ 1290.14) | 0.07 (0.03 ‒ 0.11) | 5.17 (3.44 ‒ 7.05) | 4.12 (2.78 ‒ 5.68) | -0.73 (-0.87 ‒ -0.59) |
| Costa Rica | 748.92 (580.12 ‒ 936.00) | 749.51 (583.25 ‒ 927.12) | 0.01 (-0.02 ‒ 0.04) | 2.24 (1.41 ‒ 3.22) | 2.62 (1.81 ‒ 3.62) | 0.83 (0.69 ‒ 0.97) |
| Croatia | 1253.56 (1004.10 ‒ 1521.24) | 1411.12 (1182.58 ‒ 1629.74) | 0.22 (-0.08 ‒ 0.52) | 5.13 (3.86 ‒ 6.68) | 5.45 (3.85 ‒ 7.46) | 0.57 (0.38 ‒ 0.75) |
| Cuba | 1093.39 (855.35 ‒ 1344.78) | 1386.08 (1104.01 ‒ 1723.71) | 0.95 (0.89 ‒ 1.00) | 6.99 (5.64 ‒ 8.81) | 9.36 (7.09 ‒ 12.33) | 1.31 (1.12 ‒ 1.49) |
| Cyprus | 1224.18 (953.02 ‒ 1516.51) | 1210.05 (946.15 ‒ 1518.88) | -1.38 (-2.11 ‒ -0.65) | 5.34 (3.58 ‒ 7.58) | 4.26 (2.86 ‒ 6.06) | -1.89 (-2.42 ‒ -1.36) |
| Czechia | 1699.62 (1372.39 ‒ 2054.31) | 1332.17 (1060.66 ‒ 1621.44) | 0.44 (0.03 ‒ 0.85) | 21.97 (17.10 ‒ 36.79) | 5.39 (3.97 ‒ 7.13) | -3.43 (-4.19 ‒ -2.67) |
| Cote d'Ivoire | 646.59 (508.11 ‒ 796.19) | 696.70 (546.19 ‒ 858.21) | 0.32 (0.24 ‒ 0.41) | 3.66 (2.40 ‒ 5.39) | 3.20 (2.22 ‒ 4.41) | -0.52 (-0.57 ‒ -0.46) |
| Democratic People's Republic of Korea | 966.06 (757.56 ‒ 1194.75) | 1015.16 (797.59 ‒ 1264.19) | 0.13 (0.10 ‒ 0.16) | 10.89 (5.56 ‒ 15.28) | 8.82 (4.88 ‒ 12.65) | -0.82 (-0.99 ‒ -0.66) |
| Democratic Republic of the Congo | 531.71 (416.79 ‒ 659.57) | 575.14 (446.78 ‒ 708.70) | 0.31 (0.25 ‒ 0.38) | 4.46 (2.78 ‒ 6.78) | 4.17 (2.57 ‒ 6.40) | -0.18 (-0.28 ‒ -0.08) |
| Denmark | 1286.26 (1009.26 ‒ 1595.42) | 1344.58 (1057.84 ‒ 1665.52) | 0.22 (0.17 ‒ 0.26) | 6.29 (4.85 ‒ 8.20) | 6.16 (4.71 ‒ 7.94) | 0.09 (-0.07 ‒ 0.25) |
| Djibouti | 513.15 (402.10 ‒ 628.74) | 550.04 (429.48 ‒ 677.65) | 0.30 (0.25 ‒ 0.34) | 4.72 (2.73 ‒ 7.55) | 4.88 (2.78 ‒ 8.67) | 0.02 (-0.11 ‒ 0.16) |
| Dominica | 990.98 (781.04 ‒ 1232.92) | 1103.14 (856.57 ‒ 1367.70) | 0.41 (0.38 ‒ 0.45) | 3.58 (2.47 ‒ 5.01) | 4.61 (3.34 ‒ 6.18) | 0.96 (0.90 ‒ 1.02) |
| Dominican Republic | 1007.28 (787.19 ‒ 1250.64) | 1089.59 (840.64 ‒ 1363.63) | 0.31 (0.27 ‒ 0.34) | 3.72 (2.60 ‒ 5.11) | 3.67 (2.49 ‒ 5.13) | 0.11 (-0.07 ‒ 0.30) |
| Ecuador | 1559.46 (1356.06 ‒ 1793.94) | 2023 (1868.57 ‒ 2177.03) | 1.48 (1.25 ‒ 1.71) | 5.97 (4.28 ‒ 8.03) | 7.39 (5.36 ‒ 9.68) | 1.39 (1.10 ‒ 1.69) |
| Egypt | 1144.55 (893.69 ‒ 1432.20) | 1218.21 (950.84 ‒ 1508.08) | 0.23 (0.19 ‒ 0.27) | 3.40 (2.21 ‒ 4.94) | 3.63 (2.32 ‒ 5.26) | 0.28 (0.25 ‒ 0.31) |
| El Salvador | 733.70 (569.29 ‒ 911.56) | 734.69 (576.84 ‒ 911.72) | 0.03 (-0.01 ‒ 0.06) | 2.72 (1.83 ‒ 3.86) | 2.57 (1.75 ‒ 3.56) | -0.22 (-0.34 ‒ -0.11) |
| Equatorial Guinea | 529.91 (419.92 ‒ 657.41) | 577.55 (451.96 ‒ 717.34) | 0.38 (0.31 ‒ 0.44) | 6.02 (3.30 ‒ 10.65) | 3.74 (2.41 ‒ 5.70) | -1.71 (-2.10 ‒ -1.33) |
| Eritrea | 513.22 (403.42 ‒ 633.97) | 543.6 (427.58 ‒ 670.23) | 0.23 (0.20 ‒ 0.26) | 5.71 (3.07 ‒ 10.09) | 5.57 (3.13 ‒ 9.54) | -0.02 (-0.12 ‒ 0.08) |
| Estonia | 4417.90 (3616.38 ‒ 5300.21) | 3939.19 (3090.59 ‒ 4861.57) | -0.53 (-0.65 ‒ -0.41) | 24.99 (18.28 ‒ 36.92) | 13.79 (9.78 ‒ 18.63) | -2.95 (-3.36 ‒ -2.54) |
| Eswatini | 639.36 (506.97 ‒ 792.08) | 673.04 (531.21 ‒ 827.50) | 0.20 (0.17 ‒ 0.24) | 3.75 (2.56 ‒ 5.11) | 3.90 (2.61 ‒ 5.33) | 0.36 (-0.15 ‒ 0.88) |
| Ethiopia | 611.27 (480.56 ‒ 753.66) | 601.15 (472.33 ‒ 737.84) | -0.10 (-0.15 ‒ -0.05) | 9.94 (6.32 ‒ 16.29) | 4.81 (2.97 ‒ 7.06) | -2.84 (-2.97 ‒ -2.70) |
| Fiji | 953.26 (734.48 ‒ 1194.19) | 1019.49 (784.61 ‒ 1280.35) | 0.22 (0.17 ‒ 0.27) | 3.22 (2.10 ‒ 4.63) | 3.97 (2.78 ‒ 5.46) | 1.09 (0.88 ‒ 1.30) |
| Finland | 1195.66 (915.14 ‒ 1510.17) | 1226.94 (949.83 ‒ 1534.6) | -0.60 (-1.36 ‒ 0.18) | 4.31 (2.83 ‒ 6.23) | 4.06 (2.78 ‒ 5.71) | -0.71 (-1.31 ‒ -0.11) |
| France | 1353.16 (1055.09 ‒ 1690.07) | 1371.42 (1053.58 ‒ 1713.18) | 0.05 (0.02 ‒ 0.09) | 5.48 (4.00 ‒ 7.33) | 4.80 (3.32 ‒ 6.66) | -0.43 (-0.48 ‒ -0.37) |
| Gabon | 540.89 (421.54 ‒ 668.60) | 587.82 (455.30 ‒ 728.15) | 0.36 (0.30 ‒ 0.41) | 5.58 (2.80 ‒ 10.22) | 4.78 (2.78 ‒ 7.79) | -0.56 (-0.71 ‒ -0.41) |
| Gambia | 647.26 (509.66 ‒ 786.42) | 686.49 (539.84 ‒ 841.85) | 0.26 (0.18 ‒ 0.33) | 3.49 (2.30 ‒ 5.26) | 3.40 (2.31 ‒ 4.71) | -0.17 (-0.26 ‒ -0.08) |
| Georgia | 1418.42 (1114.73 ‒ 1748.27) | 1170.08 (1070.37 ‒ 1287.75) | -0.83 (-1.00 ‒ -0.66) | 4.22 (2.74 ‒ 6.12) | 3.94 (2.85 ‒ 5.18) | -0.19 (-0.34 ‒ -0.05) |
| Germany | 1247.63 (959.57 ‒ 1568.43) | 1300.93 (1008.39 ‒ 1616.51) | 2.00 (1.36 ‒ 2.64) | 5.39 (4.05 ‒ 7.07) | 4.70 (3.27 ‒ 6.48) | 1.22 (0.75 ‒ 1.70) |
| Ghana | 897.24 (687.12 ‒ 1315.17) | 999.72 (761.04 ‒ 1545.52) | 0.34 (0.18 ‒ 0.50) | 8.61 (2.84 ‒ 16.92) | 8.35 (2.85 ‒ 13.29) | 0.32 (0.16 ‒ 0.48) |
| Greece | 1360.02 (1051.37 ‒ 1698.05) | 1370.26 (1060.65 ‒ 1712.81) | 0.06 (0.03 ‒ 0.08) | 3.98 (2.55 ‒ 5.78) | 3.99 (2.55 ‒ 5.78) | 0.09 (0.03 ‒ 0.15) |
| Greenland | 987.25 (778.35 ‒ 1212.06) | 1013.92 (800.31 ‒ 1249.00) | 0.11 (0.08 ‒ 0.15) | 3.32 (2.18 ‒ 4.56) | 3.08 (2.01 ‒ 4.43) | -0.30 (-0.41 ‒ -0.20) |
| Grenada | 1136.11 (915.69 ‒ 1400.33) | 1573.60 (1250.21 ‒ 1902.10) | 1.31 (1.20 ‒ 1.43) | 7.58 (5.95 ‒ 9.70) | 12.71 (9.73 ‒ 16.00) | 2.19 (1.96 ‒ 2.42) |
| Guam | 1050.49 (811.43 ‒ 1307.74) | 1048.85 (802.35 ‒ 1317.85) | -0.13 (-0.22 ‒ -0.04) | 7.31 (3.26 ‒ 10.20) | 3.43 (2.22 ‒ 4.89) | -3.21 (-3.93 ‒ -2.49) |
| Guatemala | 749.60 (590.26 ‒ 928.82) | 747.78 (589.19 ‒ 921.95) | -0.03 (-0.06 ‒ -0.01) | 6.52 (4.90 ‒ 8.23) | 4.97 (3.75 ‒ 6.57) | -1.07 (-1.37 ‒ -0.77) |
| Guinea | 630.97 (498.68 ‒ 772.93) | 686.68 (539.54 ‒ 843.62) | 0.23 (0.14 ‒ 0.32) | 3.88 (2.42 ‒ 6.19) | 3.44 (2.31 ‒ 4.94) | -1.18 (-1.23 ‒ -1.12) |
| Guinea-Bissau | 637.23 (499.78 ‒ 780.83) | 670.31 (526.92 ‒ 818.13) | 0.37 (0.28 ‒ 0.46) | 5.37 (3.15 ‒ 8.37) | 3.93 (2.60 ‒ 5.50) | -0.37 (-0.44 ‒ -0.30) |
| Guyana | 1068.72 (852.73 ‒ 1313.02) | 1286.55 (1037.63 ‒ 1563.12) | 0.68 (0.58 ‒ 0.78) | 7.88 (6.13 ‒ 10.64) | 11.60 (8.57 ‒ 15.11) | 1.41 (1.07 ‒ 1.75) |
| Haiti | 1010.50 (801.39 ‒ 1240.76) | 1089.33 (863.28 ‒ 1337.00) | 0.33 (0.29 ‒ 0.36) | 11.62 (6.04 ‒ 18.77) | 11.30 (6.17 ‒ 19.01) | 0.28 (0.14 ‒ 0.41) |
| Honduras | 881.65 (696.88 ‒ 1078.81) | 907.30 (716.55 ‒ 1111.34) | -0.01 (-0.15 ‒ 0.13) | 8.63 (3.92 ‒ 12.59) | 6.93 (3.98 ‒ 10.48) | -0.68 (-0.78 ‒ -0.59) |
| Hungary | 1743.52 (1412.31 ‒ 2170.94) | 1419.87 (1164.68 ‒ 1700.44) | -0.90 (-1.01 ‒ -0.79) | 28.10 (24.34 ‒ 36.87) | 9.07 (6.76 ‒ 11.68) | -3.55 (-4.18 ‒ -2.92) |
| Iceland | 1265.19 (983.89 ‒ 1575.75) | 1275.79 (995.02 ‒ 1577.90) | -0.62 (-0.84 ‒ -0.39) | 6.08 (4.72 ‒ 7.77) | 5.14 (3.68 ‒ 6.85) | -0.88 (-1.09 ‒ -0.66) |
| India | 1555.76 (1239.59 ‒ 1926.80) | 1821.01 (1434.25 ‒ 2260.91) | 0.71 (0.54 ‒ 0.88) | 12.39 (7.51 ‒ 17.97) | 8.58 (6.17 ‒ 11.43) | -1.37 (-1.57 ‒ -1.16) |
| Indonesia | 2219.91 (1747.05 ‒ 2720.30) | 1221.13 (995.99 ‒ 1451.71) | -2.79 (-3.21 ‒ -2.38) | 18.65 (6.50 ‒ 27.9) | 13.35 (4.15 ‒ 19.56) | -1.27 (-1.41 ‒ -1.13) |
| Iran (Islamic Republic of) | 1244.55 (971.16 ‒ 1551.16) | 1321.27 (1034.54 ‒ 1647.31) | 0.22 (0.15 ‒ 0.28) | 3.94 (2.61 ‒ 5.51) | 4.38 (2.97 ‒ 6.03) | 0.44 (0.26 ‒ 0.62) |
| Iraq | 1157.28 (909.38 ‒ 1432.04) | 1216.19 (956.79 ‒ 1515.10) | 0.15 (0.11 ‒ 0.19) | 5.88 (4.01 ‒ 8.73) | 4.07 (2.82 ‒ 5.62) | -1.73 (-1.95 ‒ -1.51) |
| Ireland | 1371.53 (1063.21 ‒ 1701.53) | 1376.24 (1066.09 ‒ 1717.13) | 0.04 (0.01 ‒ 0.06) | 5.69 (4.13 ‒ 7.49) | 4.91 (3.41 ‒ 6.65) | -0.50 (-0.67 ‒ -0.32) |
| Israel | 1343.34 (1035.81 ‒ 1680.34) | 1416.27 (1100.08 ‒ 1761.17) | 0.25 (0.22 ‒ 0.27) | 4.70 (3.18 ‒ 6.60) | 5.78 (4.15 ‒ 7.70) | 0.40 (0.08 ‒ 0.72) |
| Italy | 1733.39 (1357.48 ‒ 2164.50) | 1553.49 (1250.12 ‒ 1877.97) | -0.57 (-0.65 ‒ -0.49) | 7.84 (6.06 ‒ 9.94) | 5.18 (3.64 ‒ 7.00) | -1.41 (-1.66 ‒ -1.15) |
| Jamaica | 1010.25 (778.40 ‒ 1260.28) | 1165.08 (920.28 ‒ 1442.38) | 0.59 (0.56 ‒ 0.62) | 3.34 (2.24 ‒ 4.75) | 5.49 (4.02 ‒ 7.43) | 2.27 (2.04 ‒ 2.50) |
| Japan | 1579.43 (1216.08 ‒ 1973.87) | 1487.85 (1195.07 ‒ 1792.30) | -0.39 (-0.49 ‒ -0.28) | 5.51 (3.85 ‒ 7.53) | 5.97 (4.46 ‒ 7.79) | 0.39 (0.30 ‒ 0.48) |
| Jordan | 1143.80 (881.28 ‒ 1425.93) | 1792.23 (1331.12 ‒ 2622.06) | 2.10 (1.72 ‒ 2.49) | 3.75 (2.50 ‒ 5.27) | 5.19 (3.12 ‒ 8.36) | 1.54 (1.25 ‒ 1.84) |
| Kazakhstan | 2117.87 (1675.94 ‒ 2607.86) | 1947.07 (1579.25 ‒ 2360.53) | -0.63 (-0.76 ‒ -0.50) | 20.74 (14.08 ‒ 31.55) | 22.57 (17.82 ‒ 28.08) | -0.34 (-0.65 ‒ -0.02) |
| Kenya | 582.84 (456.96 ‒ 714.69) | 610.41 (479.37 ‒ 750.16) | 0.53 (0.36 ‒ 0.70) | 4.34 (2.88 ‒ 6.43) | 5.00 (3.18 ‒ 8.12) | 0.79 (0.68 ‒ 0.90) |
| Kiribati | 1017.00 (807.50 ‒ 1259.69) | 1042.86 (828.01 ‒ 1291.19) | 0.02 (0.00 ‒ 0.05) | 12.38 (6.47 ‒ 19.30) | 9.56 (5.81 ‒ 15.09) | -1.08 (-1.15 ‒ -1.02) |
| Kuwait | 1210.93 (940.48 ‒ 1519.11) | 1246.60 (959.40 ‒ 1548.12) | 0.12 (0.09 ‒ 0.14) | 3.60 (2.29 ‒ 5.27) | 3.70 (2.42 ‒ 5.40) | 0.22 (0.18 ‒ 0.26) |
| Kyrgyzstan | 1923.50 (1526.96 ‒ 2360.06) | 1907.44 (1520.62 ‒ 2345.03) | 0.81 (0.48 ‒ 1.14) | 14.10 (10.96 ‒ 18.42) | 9.15 (6.89 ‒ 11.96) | -1.37 (-1.82 ‒ -0.93) |
| Lao People's Democratic Republic | 1431.19 (1160.33 ‒ 1726.98) | 1415.83 (1131.06 ‒ 1730.50) | -0.20 (-0.26 ‒ -0.13) | 21.44 (5.12 ‒ 38.43) | 12.56 (4.37 ‒ 20.15) | -2.20 (-2.39 ‒ -2.00) |
| Latvia | 4653.72 (3823.81 ‒ 5570.62) | 4156.67 (3404.74 ‒ 5049.01) | -0.88 (-1.09 ‒ -0.67) | 29.24 (22.33 ‒ 39.45) | 20.47 (15.73 ‒ 25.88) | -2.13 (-2.46 ‒ -1.80) |
| Lebanon | 1134.76 (884.94 ‒ 1415.86) | 1202.23 (927.53 ‒ 1500.36) | 0.22 (0.19 ‒ 0.25) | 3.32 (2.11 ‒ 4.83) | 3.54 (2.24 ‒ 5.12) | 0.27 (0.23 ‒ 0.31) |
| Lesotho | 641.71 (507.23 ‒ 786.51) | 669.73 (527.81 ‒ 819.32) | 0.17 (0.11 ‒ 0.23) | 3.35 (2.21 ‒ 5.04) | 4.56 (2.93 ‒ 6.37) | 1.64 (1.36 ‒ 1.91) |
| Liberia | 644.28 (504.61 ‒ 785.79) | 694.88 (542.45 ‒ 859.58) | 0.34 (0.26 ‒ 0.42) | 4.09 (2.56 ‒ 5.92) | 3.28 (2.29 ‒ 4.53) | -0.88 (-1.05 ‒ -0.71) |
| Libya | 1173.43 (921.76 ‒ 1453.92) | 1214.85 (946.64 ‒ 1515.64) | 0.16 (0.12 ‒ 0.20) | 3.48 (2.18 ‒ 5.15) | 3.76 (2.46 ‒ 5.32) | 0.35 (0.28 ‒ 0.41) |
| Lithuania | 5022.74 (4162.80 ‒ 5935.79) | 3993.76 (3171.52 ‒ 4918.50) | -1.33 (-1.50 ‒ -1.17) | 30.39 (24.78 ‒ 37.64) | 17.20 (12.95 ‒ 22.47) | -2.87 (-3.19 ‒ -2.55) |
| Luxembourg | 1504.71 (1153.94 ‒ 1879.41) | 1558.26 (1215.2 ‒ 1963.17) | 0.06 (-0.65 ‒ 0.78) | 5.65 (4.05 ‒ 7.62) | 5.01 (3.45 ‒ 7.01) | -0.32 (-0.87 ‒ 0.23) |
| Madagascar | 514.49 (403.97 ‒ 634.66) | 535.88 (419.71 ‒ 656.23) | 0.16 (0.12 ‒ 0.21) | 5.02 (2.80 ‒ 8.51) | 4.46 (2.57 ‒ 7.56) | -0.41 (-0.49 ‒ -0.34) |
| Malawi | 515.00 (403.84 ‒ 635.84) | 538.94 (423.50 ‒ 660.74) | 0.20 (0.17 ‒ 0.23) | 5.80 (3.24 ‒ 10.19) | 4.83 (2.84 ‒ 8.01) | -0.73 (-0.81 ‒ -0.64) |
| Malaysia | 1279.74 (980.88 ‒ 1607.34) | 1400.71 (1073.18 ‒ 1746.84) | 0.33 (0.27 ‒ 0.39) | 5.41 (3.54 ‒ 7.43) | 5.70 (3.54 ‒ 9.28) | 0.25 (0.16 ‒ 0.34) |
| Maldives | 1357.43 (1048.21 ‒ 1698.81) | 1538.13 (1172.88 ‒ 1933.53) | 0.44 (0.31 ‒ 0.57) | 5.21 (3.38 ‒ 7.39) | 4.69 (3.10 ‒ 6.74) | -0.49 (-0.69 ‒ -0.29) |
| Mali | 638.39 (504.82 ‒ 780.40) | 694.13 (540.49 ‒ 852.61) | 0.32 (0.26 ‒ 0.39) | 4.74 (2.78 ‒ 7.08) | 3.52 (2.46 ‒ 4.77) | -1.09 (-1.22 ‒ -0.95) |
| Malta | 1489.40 (1154.15 ‒ 1843.73) | 1544.27 (1202.82 ‒ 1918.41) | 1.92 (1.25 ‒ 2.59) | 7.49 (5.72 ‒ 9.51) | 6.02 (4.34 ‒ 8.03) | 0.72 (0.30 ‒ 1.14) |
| Marshall Islands | 1034.16 (815.14 ‒ 1277.12) | 1073.78 (846.91 ‒ 1337.84) | 0.07 (0.05 ‒ 0.09) | 9.06 (4.75 ‒ 14.91) | 7.83 (4.57 ‒ 11.91) | -0.62 (-0.72 ‒ -0.52) |
| Mauritania | 643.10 (503.28 ‒ 783.64) | 701.94 (551.05 ‒ 861.30) | 0.33 (0.27 ‒ 0.40) | 5.15 (2.98 ‒ 7.74) | 3.35 (2.30 ‒ 4.77) | -1.59 (-1.70 ‒ -1.49) |
| Mauritius | 1292.66 (1003.38 ‒ 1627.83) | 1381.01 (1056.36 ‒ 1735.85) | 0.22 (0.18 ‒ 0.27) | 4.40 (3.09 ‒ 6.18) | 4.12 (2.70 ‒ 5.85) | -0.19 (-0.36 ‒ -0.02) |
| Mexico | 1168.03 (925.73 ‒ 1441.21) | 1226.77 (988.55 ‒ 1468.54) | 0.20 (-0.45 ‒ 0.85) | 11.73 (9.83 ‒ 13.82) | 11.41 (9.09 ‒ 15.51) | 0.00 (-0.37 ‒ 0.38) |
| Micronesia (Federated States of) | 990.65 (775.97 ‒ 1231.17) | 1043.5 (812.48 ‒ 1295.87) | 0.14 (0.12 ‒ 0.17) | 10.38 (5.39 ‒ 16.04) | 7.98 (4.61 ‒ 12.33) | -1.09 (-1.19 ‒ -1.00) |
| Monaco | 1346.68 (1033.78 ‒ 1695.42) | 1373.98 (1066.58 ‒ 1718.40) | 0.09 (0.07 ‒ 0.11) | 4.06 (2.64 ‒ 5.83) | 3.99 (2.55 ‒ 5.74) | -0.02 (-0.06 ‒ 0.01) |
| Mongolia | 1590.74 (1248.03 ‒ 1948.25) | 1658.27 (1301.87 ‒ 2047.56) | 0.19 (0.12 ‒ 0.26) | 8.19 (5.01 ‒ 12.74) | 5.66 (3.79 ‒ 7.87) | -2.01 (-2.34 ‒ -1.67) |
| Montenegro | 1280.76 (1018.60 ‒ 1562.55) | 1285.18 (1030.46 ‒ 1572.79) | 0.03 (0.00 ‒ 0.06) | 3.64 (2.35 ‒ 5.27) | 3.63 (2.36 ‒ 5.24) | 0.01 (-0.01 ‒ 0.03) |
| Morocco | 1132.73 (874.05 ‒ 1409.33) | 1201.07 (926.12 ‒ 1497.88) | 0.21 (0.16 ‒ 0.26) | 3.42 (2.19 ‒ 4.94) | 3.79 (2.47 ‒ 5.41) | 0.43 (0.38 ‒ 0.47) |
| Mozambique | 515.51 (405.23 ‒ 635.63) | 562.38 (442.82 ‒ 693.55) | 0.37 (0.34 ‒ 0.40) | 6.15 (3.26 ‒ 10.24) | 5.93 (3.21 ‒ 10.56) | 0.05 (-0.12 ‒ 0.21) |
| Myanmar | 1551.16 (1256.07 ‒ 1858.9) | 1476.21 (1184.50 ‒ 1788.06) | -0.35 (-0.44 ‒ -0.26) | 19.97 (5.54 ‒ 34.38) | 12.89 (4.63 ‒ 20.46) | -1.79 (-2.06 ‒ -1.52) |
| Namibia | 642.44 (505.03 ‒ 793.75) | 665.14 (524.36 ‒ 824.50) | 0.15 (0.09 ‒ 0.21) | 3.47 (2.33 ‒ 4.84) | 3.09 (2.17 ‒ 4.16) | -0.58 (-0.82 ‒ -0.34) |
| Nauru | 999.98 (778.49 ‒ 1242.79) | 1026.22 (789.48 ‒ 1281.90) | 0.03 (-0.02 ‒ 0.07) | 9.90 (4.80 ‒ 14.94) | 7.81 (4.42 ‒ 11.95) | -0.98 (-1.14 ‒ -0.83) |
| Nepal | 1309.13 (1033.24 ‒ 1640.25) | 1445.54 (1131.20 ‒ 1789) | 0.36 (0.30 ‒ 0.42) | 8.53 (5.44 ‒ 13.11) | 7.16 (4.85 ‒ 10.59) | -0.51 (-0.78 ‒ -0.25) |
| Netherlands | 1518.48 (1195.86 ‒ 1888.49) | 1448.71 (1133.74 ‒ 1803.40) | -0.24 (-0.27 ‒ -0.20) | 9.29 (7.44 ‒ 11.47) | 6.21 (4.59 ‒ 8.04) | -1.51 (-1.70 ‒ -1.32) |
| New Zealand | 1655.45 (1296.13 ‒ 2071.05) | 1288.37 (1082.49 ‒ 1508.08) | -0.83 (-1.06 ‒ -0.60) | 10.29 (8.19 ‒ 13.07) | 7.30 (5.82 ‒ 9.03) | -0.90 (-1.30 ‒ -0.51) |
| Nicaragua | 735.36 (572.89 ‒ 913.31) | 749.44 (582.73 ‒ 924.68) | 0.11 (0.07 ‒ 0.14) | 3.11 (2.16 ‒ 4.26) | 3.68 (2.34 ‒ 5.01) | 0.51 (0.20 ‒ 0.81) |
| Niger | 639.89 (503.84 ‒ 781.72) | 683.57 (536.70 ‒ 838.42) | 0.29 (0.21 ‒ 0.37) | 4.37 (2.75 ‒ 6.63) | 3.30 (2.22 ‒ 4.49) | -1.20 (-1.32 ‒ -1.09) |
| Nigeria | 706.16 (558.87 ‒ 868.67) | 732.17 (579.07 ‒ 897.20) | 0.18 (0.10 ‒ 0.26) | 3.53 (2.39 ‒ 4.93) | 2.98 (2.13 ‒ 4.00) | -0.69 (-0.77 ‒ -0.62) |
| Niue | 971.82 (751.37 ‒ 1211.08) | 1047.22 (808.55 ‒ 1311.31) | 0.24 (0.22 ‒ 0.26) | 6.47 (3.80 ‒ 9.05) | 5.15 (3.33 ‒ 7.15) | -1.01 (-1.08 ‒ -0.93) |
| North Macedonia | 1280.32 (1026.85 ‒ 1566.27) | 1289.99 (1034.39 ‒ 1578.54) | 0.05 (0.02 ‒ 0.08) | 3.83 (2.52 ‒ 5.38) | 3.67 (2.35 ‒ 5.27) | -0.12 (-0.19 ‒ -0.05) |
| Northern Mariana Islands | 1058.48 (815.58 ‒ 1321.94) | 1061.68 (814.60 ‒ 1327) | -0.02 (-0.05 ‒ 0.02) | 5.45 (3.81 ‒ 7.63) | 3.78 (2.56 ‒ 5.44) | -1.14 (-1.47 ‒ -0.81) |
| Norway | 2605.46 (2057.64 ‒ 3211.68) | 2537.56 (1985.32 ‒ 3154.17) | 0.64 (0.41 ‒ 0.87) | 11.39 (8.70 ‒ 14.43) | 8.64 (6.10 ‒ 11.64) | -0.21 (-0.44 ‒ 0.01) |
| Oman | 1210.34 (940.70 ‒ 1520.44) | 1309.32 (1014.21 ‒ 1643.65) | 0.31 (0.24 ‒ 0.38) | 3.73 (2.42 ‒ 5.43) | 4.18 (2.72 ‒ 6.04) | 0.64 (0.54 ‒ 0.75) |
| Pakistan | 1410.40 (1109.74 ‒ 1752.55) | 1542.81 (1202.33 ‒ 1922.15) | 0.31 (0.22 ‒ 0.41) | 9.43 (6.20 ‒ 13.89) | 9.34 (6.61 ‒ 13.24) | -0.24 (-0.41 ‒ -0.06) |
| Palau | 991.70 (766.12 ‒ 1233.37) | 1083.69 (841.06 ‒ 1347.36) | 0.23 (0.18 ‒ 0.28) | 6.35 (3.72 ‒ 9.28) | 5.39 (3.37 ‒ 8.05) | -0.62 (-0.67 ‒ -0.57) |
| Palestine | 1123.17 (868.15 ‒ 1405.33) | 1208.65 (943.36 ‒ 1513.20) | 0.28 (0.25 ‒ 0.31) | 4.08 (2.72 ‒ 5.75) | 3.88 (2.55 ‒ 5.55) | -0.19 (-0.30 ‒ -0.07) |
| Panama | 749.55 (581.96 ‒ 932.81) | 754.15 (581.71 ‒ 936.92) | 0.02 (-0.01 ‒ 0.05) | 2.38 (1.59 ‒ 3.39) | 2.49 (1.69 ‒ 3.48) | 0.25 (0.06 ‒ 0.45) |
| Papua New Guinea | 973.82 (755.39 ‒ 1215.32) | 1031.41 (795.86 ‒ 1294.47) | 0.16 (0.10 ‒ 0.21) | 7.70 (3.72 ‒ 11.66) | 6.42 (3.61 ‒ 9.62) | -0.65 (-0.70 ‒ -0.60) |
| Paraguay | 943.67 (738.08 ‒ 1163.44) | 961.65 (759.04 ‒ 1188.35) | 0.08 (0.06 ‒ 0.10) | 3.65 (2.46 ‒ 4.97) | 4.76 (2.78 ‒ 6.69) | 1.25 (0.94 ‒ 1.57) |
| Peru | 1635.69 (1264.27 ‒ 2071.98) | 1684.65 (1297.75 ‒ 2127.67) | 0.14 (0.09 ‒ 0.18) | 5.76 (3.86 ‒ 8.09) | 5.53 (3.70 ‒ 7.76) | -0.07 (-0.17 ‒ 0.04) |
| Philippines | 2365.26 (1900.48 ‒ 2848.35) | 2696.74 (2177.83 ‒ 3255.03) | 0.40 (0.24 ‒ 0.57) | 26.90 (19.70 ‒ 34.07) | 22.66 (18.14 ‒ 29.72) | -0.66 (-0.76 ‒ -0.57) |
| Poland | 2112.96 (1642.61 ‒ 2634.18) | 873.44 (762.46 ‒ 999.21) | -3.87 (-4.49 ‒ -3.24) | 15.34 (12.61 ‒ 19.47) | 3.33 (2.49 ‒ 4.41) | -5.84 (-6.33 ‒ -5.35) |
| Portugal | 1180.14 (918.40 ‒ 1476.43) | 1249.49 (1091.66 ‒ 1438.16) | 0.30 (0.26 ‒ 0.35) | 4.73 (3.34 ‒ 6.42) | 4.87 (3.37 ‒ 6.84) | 0.38 (0.08 ‒ 0.67) |
| Puerto Rico | 1012.68 (781.72 ‒ 1269.84) | 1087.25 (840.82 ‒ 1357.17) | 0.29 (0.26 ‒ 0.32) | 3.04 (1.93 ‒ 4.41) | 3.65 (2.51 ‒ 5.17) | 0.65 (0.54 ‒ 0.76) |
| Qatar | 1251.05 (975.44 ‒ 1570.60) | 1363.09 (1062.01 ‒ 1706.32) | 0.38 (0.34 ‒ 0.42) | 3.86 (2.45 ‒ 5.64) | 4.00 (2.58 ‒ 5.95) | 0.27 (0.21 ‒ 0.34) |
| Republic of Korea | 1397.08 (1078.97 ‒ 1754.82) | 1443.72 (1109.59 ‒ 1807.66) | 0.10 (0.06 ‒ 0.14) | 5.37 (3.70 ‒ 7.39) | 4.99 (3.41 ‒ 7.02) | -0.40 (-0.55 ‒ -0.25) |
| Republic of Moldova | 3746.50 (2984.79 ‒ 4570.33) | 3918.49 (3057.48 ‒ 4877.04) | 0.31 (0.24 ‒ 0.38) | 17.84 (13.67 ‒ 22.8) | 17.04 (12.47 ‒ 22.23) | 0.01 (-0.34 ‒ 0.36) |
| Romania | 1350.93 (1075.78 ‒ 1655.13) | 1354.04 (1075.96 ‒ 1656.63) | 2.01 (1.34 ‒ 2.69) | 3.95 (2.59 ‒ 5.60) | 3.92 (2.55 ‒ 5.52) | 1.86 (1.24 ‒ 2.49) |
| Russian Federation | 5238.69 (4218.41 ‒ 6314.79) | 4541.88 (3648.94 ‒ 5522) | -0.63 (-0.80 ‒ -0.45) | 31.36 (25.72 ‒ 38.41) | 24.65 (19.66 ‒ 30.59) | -1.06 (-1.39 ‒ -0.73) |
| Rwanda | 525.06 (410.46 ‒ 643.69) | 542.34 (427.05 ‒ 670.24) | 0.13 (0.08 ‒ 0.18) | 6.37 (1.67 ‒ 12.37) | 4.48 (1.69 ‒ 9.06) | -1.87 (-2.14 ‒ -1.60) |
| Saint Kitts and Nevis | 1028.96 (809.01 ‒ 1264.60) | 1121.98 (879.96 ‒ 1389.75) | 0.31 (0.29 ‒ 0.32) | 4.54 (3.34 ‒ 6.04) | 5.03 (3.59 ‒ 6.72) | 0.24 (0.12 ‒ 0.37) |
| Saint Lucia | 1051.09 (826.03 ‒ 1296.45) | 1187.62 (933.68 ‒ 1471.61) | 0.47 (0.45 ‒ 0.49) | 6.11 (4.83 ‒ 7.73) | 7.97 (6.14 ‒ 10.18) | 0.84 (0.69 ‒ 0.99) |
| Saint Vincent and the Grenadines | 1086.5 (858.72 ‒ 1337.62) | 1276.00 (1014.06 ‒ 1571.81) | 0.65 (0.61 ‒ 0.69) | 6.33 (4.80 ‒ 8.41) | 7.75 (5.76 ‒ 9.87) | 0.93 (0.72 ‒ 1.13) |
| Samoa | 1006.99 (785.02 ‒ 1246.56) | 1067.93 (827.24 ‒ 1324.48) | 0.13 (0.10 ‒ 0.16) | 8.06 (4.38 ‒ 11.57) | 6.14 (3.82 ‒ 8.74) | -1.20 (-1.30 ‒ -1.10) |
| San Marino | 1427.11 (1119.41 ‒ 1790.96) | 1413.92 (1098.95 ‒ 1759.89) | -0.06 (-0.08 ‒ -0.05) | 7.01 (5.09 ‒ 9.66) | 7.27 (5.29 ‒ 10.14) | 0.46 (0.30 ‒ 0.61) |
| Sao Tome and Principe | 634.07 (505.44 ‒ 778.77) | 696.66 (549.24 ‒ 858.51) | 0.36 (0.30 ‒ 0.42) | 3.79 (2.46 ‒ 5.39) | 3.63 (2.25 ‒ 5.69) | -0.43 (-0.57 ‒ -0.29) |
| Saudi Arabia | 1198.47 (934.96 ‒ 1496.56) | 1273.28 (989.02 ‒ 1597.24) | 0.22 (0.19 ‒ 0.26) | 3.52 (2.21 ‒ 5.15) | 3.69 (2.36 ‒ 5.35) | 0.23 (0.21 ‒ 0.26) |
| Senegal | 636.32 (500.79 ‒ 784.54) | 683.92 (535.09 ‒ 841.96) | 0.32 (0.24 ‒ 0.40) | 4.14 (2.60 ‒ 6.13) | 3.24 (2.28 ‒ 4.50) | -0.91 (-1.02 ‒ -0.81) |
| Serbia | 1224.07 (975.78 ‒ 1491.42) | 1245.04 (993.33 ‒ 1518.06) | 1.63 (1.11 ‒ 2.16) | 4.81 (3.44 ‒ 6.53) | 4.48 (3.14 ‒ 6.03) | 0.87 (0.49 ‒ 1.25) |
| Seychelles | 1313.35 (1038.67 ‒ 1642.68) | 1509.98 (1197.97 ‒ 1850.35) | 0.47 (0.42 ‒ 0.52) | 6.85 (3.48 ‒ 9.66) | 9.73 (4.17 ‒ 14.71) | 1.04 (0.79 ‒ 1.28) |
| Sierra Leone | 634.26 (501.13 ‒ 775.24) | 689.39 (545.14 ‒ 853.67) | 0.36 (0.27 ‒ 0.45) | 3.51 (2.32 ‒ 4.99) | 3.20 (2.22 ‒ 4.46) | -0.26 (-0.30 ‒ -0.22) |
| Singapore | 1475.38 (1151.98 ‒ 1845.83) | 1460 (1121.81 ‒ 1834.12) | -0.02 (-0.09 ‒ 0.04) | 6.91 (5.19 ‒ 9.05) | 4.48 (2.90 ‒ 6.47) | -1.09 (-1.44 ‒ -0.73) |
| Slovakia | 1442.90 (1158.93 ‒ 1747.18) | 1416.67 (1127.33 ‒ 1728.94) | 0.23 (-0.60 ‒ 1.07) | 8.86 (5.91 ‒ 11.52) | 5.20 (3.67 ‒ 7.14) | -1.43 (-1.85 ‒ -1.00) |
| Slovenia | 1210.66 (967.73 ‒ 1467.35) | 1212.30 (964.10 ‒ 1480.70) | 0.70 (0.18 ‒ 1.22) | 5.90 (4.04 ‒ 8.90) | 3.92 (2.62 ‒ 5.45) | -0.91 (-1.24 ‒ -0.59) |
| Solomon Islands | 1023.80 (806.72 ‒ 1266.95) | 1050.73 (819.23 ‒ 1312.54) | 0.01 (-0.02 ‒ 0.04) | 10.70 (5.64 ‒ 16.26) | 9.04 (5.06 ‒ 14.12) | -0.56 (-0.66 ‒ -0.45) |
| Somalia | 525.29 (412.33 ‒ 642.65) | 542.22 (422.76 ‒ 665.05) | 0.13 (0.11 ‒ 0.16) | 7.45 (3.51 ‒ 13.82) | 7.56 (3.37 ‒ 14.06) | 0.21 (0.09 ‒ 0.32) |
| South Africa | 714.12 (561.63 ‒ 879.16) | 740.36 (584.86 ‒ 910.43) | 0.12 (0.07 ‒ 0.17) | 3.84 (2.91 ‒ 4.92) | 3.33 (2.48 ‒ 4.27) | -0.49 (-0.89 ‒ -0.09) |
| South Sudan | 497.78 (390.80 ‒ 614.51) | 533.43 (416.22 ‒ 657.46) | 0.26 (0.22 ‒ 0.31) | 4.29 (2.39 ‒ 7.28) | 3.96 (2.33 ‒ 6.54) | -0.35 (-0.46 ‒ -0.24) |
| Spain | 1351.29 (1058.79 ‒ 1690.45) | 1388.66 (1084.79 ‒ 1732.65) | 0.12 (0.10 ‒ 0.14) | 5.57 (4.08 ‒ 7.36) | 4.88 (3.37 ‒ 6.74) | -0.37 (-0.47 ‒ -0.27) |
| Sri Lanka | 1321.85 (1015.11 ‒ 1647.53) | 1354.93 (1049.65 ‒ 1685.03) | 0.09 (0.02 ‒ 0.15) | 3.92 (2.58 ‒ 5.68) | 4.07 (2.67 ‒ 5.82) | 0.17 (0.11 ‒ 0.23) |
| Sudan | 1125.89 (870.78 ‒ 1407.81) | 1193.14 (927.08 ‒ 1475.74) | 0.22 (0.18 ‒ 0.26) | 3.50 (2.26 ‒ 5.14) | 3.83 (2.53 ‒ 5.47) | 0.43 (0.37 ‒ 0.49) |
| Suriname | 1148.04 (915.69 ‒ 1402.14) | 1429.08 (1150.25 ‒ 1721.64) | 0.88 (0.76 ‒ 1.00) | 7.40 (5.41 ‒ 10.42) | 11.95 (7.71 ‒ 16.16) | 1.65 (1.32 ‒ 1.98) |
| Sweden | 1541.09 (1200.52 ‒ 1894.11) | 1348.10 (1057.00 ‒ 1661.90) | -0.14 (-0.43 ‒ 0.15) | 7.27 (5.52 ‒ 9.25) | 5.43 (4.02 ‒ 7.14) | -0.47 (-0.91 ‒ -0.04) |
| Switzerland | 1477.16 (1144.37 ‒ 1842.46) | 1503.18 (1160.40 ‒ 1867.68) | 0.24 (-0.17 ‒ 0.66) | 5.67 (4.04 ‒ 7.63) | 4.81 (3.19 ‒ 6.79) | -0.34 (-0.60 ‒ -0.07) |
| Syrian Arab Republic | 1138.87 (884.63 ‒ 1409.61) | 1194.85 (934.41 ‒ 1495.66) | 0.21 (0.18 ‒ 0.23) | 4.04 (2.65 ‒ 5.84) | 3.67 (2.39 ‒ 5.34) | -0.47 (-0.56 ‒ -0.39) |
| Taiwan (Province of China) | 1094.80 (853.40 ‒ 1363.14) | 1453.00 (1222.94 ‒ 1713.33) | 1.07 (0.76 ‒ 1.38) | 5.88 (4.43 ‒ 7.64) | 6.82 (5.02 ‒ 8.95) | 1.39 (0.64 ‒ 2.16) |
| Tajikistan | 1612.29 (1275.62 ‒ 1970.71) | 1742.18 (1401.67 ‒ 2129.84) | 0.29 (0.26 ‒ 0.33) | 9.01 (5.77 ‒ 12.04) | 11.56 (7.72 ‒ 15.43) | 0.74 (0.55 ‒ 0.92) |
| Thailand | 1824.82 (1468.04 ‒ 2202.06) | 1608.40 (1301.43 ‒ 1968.53) | -0.76 (-0.94 ‒ -0.58) | 19.59 (9.28 ‒ 29.00) | 12.10 (6.37 ‒ 18.34) | -2.36 (-2.92 ‒ -1.80) |
| Timor-Leste | 1306.52 (1045.35 ‒ 1613.49) | 1370.39 (1085.12 ‒ 1697.64) | 0.16 (0.10 ‒ 0.22) | 13.31 (4.09 ‒ 25.49) | 11.72 (4.11 ‒ 19.36) | -0.59 (-0.77 ‒ -0.40) |
| Togo | 630.75 (502.91 ‒ 769.46) | 665.35 (525.65 ‒ 817.76) | 0.25 (0.16 ‒ 0.34) | 3.87 (2.46 ‒ 5.87) | 3.21 (2.22 ‒ 4.47) | -0.78 (-0.86 ‒ -0.69) |
| Tokelau | 931.89 (722.79 ‒ 1160.20) | 1035.34 (798.73 ‒ 1292.42) | 0.35 (0.31 ‒ 0.40) | 7.89 (4.13 ‒ 12.06) | 5.55 (3.56 ‒ 7.79) | -1.39 (-1.48 ‒ -1.31) |
| Tonga | 952.95 (748.19 ‒ 1187.94) | 1029.01 (801.66 ‒ 1287.89) | 0.24 (0.21 ‒ 0.27) | 5.85 (3.57 ‒ 8.57) | 5.37 (3.56 ‒ 7.50) | -0.41 (-0.52 ‒ -0.30) |
| Trinidad and Tobago | 1271.59 (983.96 ‒ 1570.85) | 1768.54 (1338.69 ‒ 2882.31) | 1.52 (1.29 ‒ 1.75) | 11.00 (8.63 ‒ 15.45) | 18.05 (12.95 ‒ 26.14) | 2.84 (2.28 ‒ 3.41) |
| Tunisia | 1143.41 (884.55 ‒ 1418.63) | 1212.34 (946.37 ‒ 1517.71) | 0.20 (0.17 ‒ 0.23) | 3.37 (2.11 ‒ 4.83) | 3.59 (2.33 ‒ 5.18) | 0.24 (0.21 ‒ 0.27) |
| Turkey | 1132.32 (891.87 ‒ 1412.05) | 1232.54 (971.68 ‒ 1540.71) | 0.34 (0.31 ‒ 0.37) | 4.77 (3.30 ‒ 6.63) | 4.55 (3.22 ‒ 6.24) | 0.07 (-0.10 ‒ 0.23) |
| Turkmenistan | 1548.21 (1218.54 ‒ 1895.93) | 1668.62 (1315.54 ‒ 2051.08) | 0.31 (0.23 ‒ 0.40) | 5.84 (4.10 ‒ 8.06) | 8.95 (6.79 ‒ 11.48) | 1.79 (1.64 ‒ 1.94) |
| Tuvalu | 923.27 (715.25 ‒ 1155.93) | 1035.87 (800.02 ‒ 1296.82) | 0.35 (0.31 ‒ 0.40) | 9.39 (4.71 ‒ 15.32) | 6.24 (3.95 ‒ 9.33) | -1.45 (-1.54 ‒ -1.36) |
| Uganda | 506.91 (396.42 ‒ 625.03) | 536.57 (417.29 ‒ 660.79) | 0.21 (0.17 ‒ 0.25) | 3.85 (1.79 ‒ 6.56) | 4.04 (2.04 ‒ 6.82) | -0.10 (-0.30 ‒ 0.11) |
| Ukraine | 5185.73 (4190.92 ‒ 6278.78) | 4282.60 (3377.63 ‒ 5271.76) | -0.94 (-1.11 ‒ -0.78) | 26.20 (20.66 ‒ 32.85) | 22.63 (17.36 ‒ 28.82) | -1.07 (-1.32 ‒ -0.81) |
| United Arab Emirates | 1247.16 (953.67 ‒ 1567.20) | 1337.56 (1047.60 ‒ 1680.12) | 0.28 (0.25 ‒ 0.31) | 3.87 (2.49 ‒ 5.71) | 4.28 (2.71 ‒ 6.17) | 0.55 (0.46 ‒ 0.65) |
| United Kingdom | 1645.59 (1282.38 ‒ 2047.69) | 1792.30 (1447.42 ‒ 2165.2) | 0.83 (0.60 ‒ 1.06) | 7.76 (5.74 ‒ 9.98) | 8.21 (6.29 ‒ 10.27) | 0.65 (0.40 ‒ 0.91) |
| United Republic of Tanzania | 509.95 (399.86 ‒ 624.39) | 540.14 (422.76 ‒ 658.60) | 0.23 (0.18 ‒ 0.28) | 4.41 (2.55 ‒ 7.29) | 4.19 (2.45 ‒ 7.31) | -0.10 (-0.23 ‒ 0.02) |
| United States of America | 1690.06 (1325.57 ‒ 2098.28) | 974.46 (844.52 ‒ 1120.12) | -2.25 (-2.60 ‒ -1.89) | 6.36 (4.68 ‒ 8.35) | 4.49 (3.55 ‒ 5.58) | -1.19 (-1.49 ‒ -0.88) |
| United States Virgin Islands | 1210.10 (963.56 ‒ 1474.27) | 1571.62 (1217.85 ‒ 1928.31) | 1.19 (0.92 ‒ 1.46) | 12.53 (9.29 ‒ 18.71) | 16.28 (12.06 ‒ 21.55) | 1.39 (1.16 ‒ 1.63) |
| Uruguay | 1639.67 (1263.3 ‒ 2080.34) | 1676.55 (1305.44 ‒ 2117.06) | 0.10 (0.07 ‒ 0.14) | 5.29 (3.56 ‒ 7.48) | 6.03 (4.23 ‒ 8.25) | 0.54 (0.47 ‒ 0.62) |
| Uzbekistan | 1592.65 (1242.96 ‒ 1967.21) | 1679.65 (1334.46 ‒ 2080.39) | 0.21 (0.15 ‒ 0.26) | 4.51 (2.85 ‒ 6.57) | 4.84 (3.10 ‒ 6.96) | 0.29 (0.25 ‒ 0.34) |
| Vanuatu | 961.91 (741.15 ‒ 1194.00) | 1018.25 (774.90 ‒ 1274.89) | 0.17 (0.11 ‒ 0.23) | 7.55 (4.07 ‒ 11.54) | 7.03 (4.21 ‒ 11.22) | -0.43 (-0.56 ‒ -0.31) |
| Venezuela (Bolivarian Republic of) | 831.17 (653.94 ‒ 1019.67) | 860.12 (684.06 ‒ 1054.01) | 0.02 (-0.07 ‒ 0.12) | 6.09 (4.82 ‒ 8.49) | 8.27 (6.17 ‒ 10.95) | 0.39 (-0.22 ‒ 1.00) |
| Viet Nam | 1368.93 (1079.00 ‒ 1693.70) | 2074.64 (1579.98 ‒ 2770.63) | 1.85 (1.53 ‒ 2.17) | 7.85 (3.39 ‒ 11.76) | 8.52 (4.68 ‒ 12.40) | 0.51 (0.16 ‒ 0.86) |
| Yemen | 1127.75 (878.28 ‒ 1407.55) | 1198.72 (930.36 ‒ 1489.50) | 0.22 (0.17 ‒ 0.26) | 3.45 (2.15 ‒ 5.13) | 3.86 (2.53 ‒ 5.54) | 0.47 (0.41 ‒ 0.53) |
| Zambia | 533.17 (421.02 ‒ 655.07) | 542.89 (426.97 ‒ 666.88) | 0.05 (0.01 ‒ 0.10) | 6.34 (3.66 ‒ 11.15) | 5.26 (2.98 ‒ 9.66) | -0.86 (-0.98 ‒ -0.74) |
| Zimbabwe | 678.92 (538.53 ‒ 830.83) | 687.19 (547.16 ‒ 846.74) | 0.00 (-0.02 ‒ 0.02) | 3.21 (2.11 ‒ 4.38) | 3.70 (2.35 ‒ 5.27) | 0.52 (0.18 ‒ 0.86) |

DALYs, disability-adjusted life years; ASR, age-standardized rate; EAPC, estimated annual percentage change; UI, uncertainty interval.
